# Complexity of cardiac signals for predicting changes in alpha-waves after stress in patients undergoing cardiac catheterization

**DOI:** 10.1038/srep13315

**Published:** 2015-08-19

**Authors:** Hung-Chih Chiu, Yen-Hung Lin, Men-Tzung Lo, Sung-Chun Tang, Tzung-Dau Wang, Hung-Chun Lu, Yi-Lwun Ho, Hsi-Pin Ma, Chung-Kang Peng

**Affiliations:** 1 Department of Electrical Engineering, National Tsing Hua University, Hsinchu, Taiwan; 2 Department of Internal Medicine, National Taiwan University Hospital and National Taiwan University College of Medicine, Taipei, Taiwan; 3 Institute of Translational and Interdisciplinary Medicine and Department of Biomedical Sciences and Engineering, National Central University, Chungli, Taiwan; 4 Research Center for Adaptive Data Analysis, National Central University, Taoyuan, Taiwan; 5 Division of Interdisciplinary Medicine and Biotechnology, Beth Israel Deaconess Medical Center/Harvard Medical School, Boston, Massachusetts, USA; 6 Department of Neurology, National Taiwan University Hospital and National Taiwan University College of Medicine, Taipei, Taiwan

## Abstract

The hierarchical interaction between electrical signals of the brain and heart is not fully understood. We hypothesized that the complexity of cardiac electrical activity can be used to predict changes in encephalic electricity after stress. Most methods for analyzing the interaction between the heart rate variability (HRV) and electroencephalography (EEG) require a computation-intensive mathematical model. To overcome these limitations and increase the predictive accuracy of human relaxing states, we developed a method to test our hypothesis. In addition to routine linear analysis, multiscale entropy and detrended fluctuation analysis of the HRV were used to quantify nonstationary and nonlinear dynamic changes in the heart rate time series. Short-time Fourier transform was applied to quantify the power of EEG. The clinical, HRV, and EEG parameters of postcatheterization EEG alpha waves were analyzed using change-score analysis and generalized additive models. In conclusion, the complexity of cardiac electrical signals can be used to predict EEG changes after stress.

The relationship between the human brain and human heart is comparable to that between a rider and a horse. Using a bridle, the rider controls the pace of his horse, whether trotting or cantering. Conversely, by observing the pace at which the horse moves, onlookers can deduce the method that the rider uses to control the horse as well as his thought processes. It is generally believed that a human being’s heart and brain are hierarchically connected such that the heart receives the brain’s commands through a central autonomic network^1^. The autonomic nervous system (ANS) correlates with the physiological and pathological states^2^,^3^,^4^, in which changes typically reflect the heartbeat and brainwaves that communicate through regulatory central nervous system (CNS) signals. Because electroencephalography (EEG) alterations affect the physiological response pattern, the use of EEG has been proposed in practical applications to evaluate physiological responses^5^,^6^. In addition to EEG, a methodology for identifying relaxing states based on patterns from the heart rate and skin conductance has been investigated over the past decade^7^,^8^. However, medication and environmental factors may interfere with the results obtained for the skin conductance and heart rate. Hence, machine learning with the capacity to select features associated with physiological and pathological states has been used to identify emotion recognition systems^9^,^10^. According to methods that are acceptable in the field, the clinical applications of these measurements are limited by their multiple signals and complex mathematical models for physiological signals.

Compared with the previously proposed models, this study used a regression model and machine learning technique, which are referred to as change score analysis and generalized additive models, to evaluate relaxing states. These techniques require purely parametric values that are associated with the heart and brain. Our aim was to determine the optimal fitting of change score analysis using the difference between the pretest and post-test values obtained for electrocardiography (ECG) and EEG signals.

Researchers have begun assessing the human physiological states and ANS because ANS activity can be easily monitored through wearable sensors^11^. Hence, heart rate variability (HRV) analyses can be used to noninvasively assess autonomic function. Time- and frequency-based metrics^12^,^13^ have been demonstrated to have prognostic value and can be linked to other clinical or biological prognostic metrics^14^,^15^,^16^. However, conventional linear HRV analyses, which are based on both time and frequency analysis, have several limitations due to the complex autonomic function of heartbeat dynamics, which consist of interconnected feedback loops^17^. For instance, although numerous researchers have performed a spectral estimation of biomedical signals, this estimate cannot sufficiently display the nonlinear and nonstationary properties of complex biological systems^12^,^18^.

Recently, the sciences of complexity and classification algorithms have been closely related to HRV analysis, which characterizes nonlinear dynamics^19^,^20^. The present study investigated heartbeat features using two innovative analysis methods that are derived from nonlinear and nonstationary processes. The first method was a multiscale entropy (MSE) measurement method^21^, which was applied using a nonlinear algorithm that provides the regularity pattern of a time series by analyzing the interplay between quantitative connotations and the correlations among individual subjects. The second method is called detrended fluctuation analysis (DFA)^22^ and is used to evaluate the fractal correlation that causes a heart rate fluctuation originating from the interactive regulatory mechanisms. In addition, the short-term (∝_1_) and long-term (∝_2_) correlation exponents derived from the DFA analysis of heartbeat time series were calculated to clarify the fractal correlation property in a physiological system^23^.

Although previous studies have yielded considerable valuable information that can be used to assess the human physiological states, many of the factors that can predict relaxation remain unexplored. The changes in the relaxing state of energy in EEG alpha bands are a critical indicator of the processes^24^,^25^ associated with brain waves. Fourier-based spectral analysis has been widely used to investigate the spectra of EEG signals. However, the conventional Fourier transform only provides a coarse frequency estimate, which is unsuitable for short wavelengths. Hence, time-frequency transforms are used to calculate the alpha activity from clinical EEG data, and numerous methods are thus applied to estimate the time–frequency density of biomedical signals^26^,^27^. In particular, a short time window is applied to biomedical signals, and Fourier transforms are performed within this window as it slides across the range of data. This technique was incorporated in the change score analysis that was proposed in the present research, which could accurately predict people’s relaxing states.

During the embryonic stage, the heart begins to beat before the brain is formed. It is not completely understood how the electrical signals between the brain and heart interact hierarchically. In this study, we tested the hypothesis that the complexity of cardiac electric activity can be used to predict the changes in encephalic electricity after stress. We adopted linear and nonlinear cardiac electrical activity features and the parameters associated with EEG alpha bands to recognize relaxing states.

## Results

### Study patient demographics

A total of 117 patients were enrolled in the study. All patients tolerated the procedure well, and no clinical complications were noted during the index procedure or admission. Among them, thirty-three patients were excluded because of poor data recording and incomplete EEG recording, and the remaining 84 patients were selected for the final analysis. The clinical information of all patients (n = 84) are presented in Table 1. The mean age was 64.1 ± 11.8 years, and 69 were men. Sixty-five patients underwent both cardiac catheterization and coronary artery intervention (including stent implantation) in the index procedure (revascularization group). Nineteen patients underwent cardiac catheterization alone (nonrevascularization group) owing to the lack of significant stenosis in the index procedure.

### Serum neurotransmitter measurement and ECG/EEG parameters in the revascularization and nonrevascularization groups

The differences in the serum neurotransmitter levels, HRV metrics and EEG parameters are presented in Table 2. In terms of serum neurotransmitters, the dopamine level was high in the revascularization group (231.60 ± 224.09 vs. 148.96 ± 201.66; *p* = 0.047). The differences in the orphanin FQ and serotonin levels between the groups were not statistically significant. In the EEG reading, the pre-procedure alpha activity, post-procedure alpha activity, and difference in the pre/post procedure alpha activity were comparable in both groups. The HRV metrics of pNN20 (pre-) and log-pNN20 (pre-) in the revascularization group were significantly different (0.4974 ± 0.2553 vs. 0.3567 ± 0.2267.66; *p* = 0.026 and −0.87 ± 0.68 vs. −1.27 ± 0.77; *p* = 0.026). The other HRV parameters were comparable in both groups.

### Experimental framework

**Figure 1:**
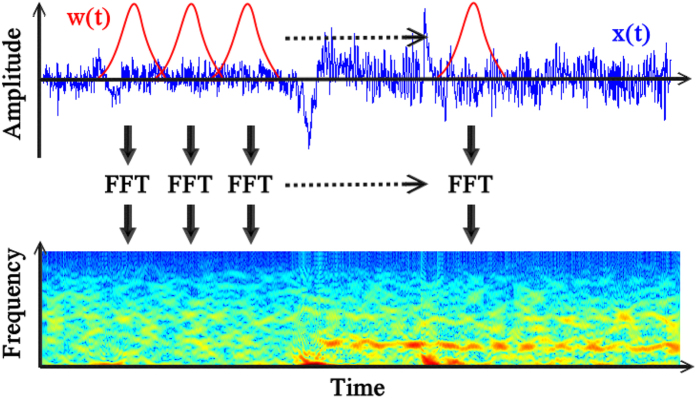
Overview of the experimental design for signal processing and statistical analysis. (**a**,**b**) Conventional HRV metrics were calculated in the time and frequency domains. (**c**) STFT is a type of spectral analysis with a fixed-width window and yields an instantaneous estimate of the time-varying energy. (**d**) Clinical information on the control and coronary artery disease (CAD) patients. (**e**) The continuous ECG and EEG parameters were analyzed using Spearman’s rank correlation to remove confounding variables. (**f**,**g**) Statistical analyses were performed using the stepwise variable selection method, change-score analysis, and GAMs.

Figure 1 shows the proposed framework, which consists of multiple analysis steps, including an overall variation analysis method (time- and frequency-based analysis), statistical method, and clinical data. Each ECG and EEG segment, with a time series of 4 min, was used for signal processing and statistical analysis. The novel ideal of the procedure is emphasized, and details of the procedure are presented in the Methods section. This procedure is completely based on the linear and nonlinear analysis of the pre- and post-test heartbeat. To improve the procedure and enhance the accuracy of predicting human’s relaxing states, we included the post-catheterization EEG alpha waves and clinical data. In particular, we used STFT when there was a change in the encephalic electricity.

The statistics on the heartbeat and brainwaves were obtained using change-score analysis. Before model fitting, the continuous ECG and EEG parameters were analyzed using Spearman’s rank correlation to remove a confounding variable, and GAMs were used to increase the prediction accuracy.

### Data classification and definitions

ECG parameters were computed using two metrics, i.e., linear and nonlinear methods, as shown in Supplementary Table S1. Some HRV metrics showed strong interactions between HRV and EEG, with a correlation coefficient of 0.8 (see Supplementary Table S4 and S5 for details). The correlation between MSE and conventional HRV analysis was nonsignificant for all patients. The EEG alpha activity was determined using STFT. The experiment involved the use of 52 continuous variables to investigate the role of relaxing states (post-test alpha activity). To enhance personalization and improve the investigation accuracy, the change score analysis derived 11 discrete variables of clinical data and 3 continuous serum neurotransmitter variables.

Before multiple regression model fitting, the continuous parameters of ECG and EEG were analyzed using Spearman’s rank correlation to remove confounding variables from the test for continuous variables, which resulted in 23 continuous variables. Supplementary Table S2 shows the pretest, and the difference between the pre- and post-test was considered after determining the Spearman’s rank correlation. To determine the prognostic value for the recognition of relaxed states, we proposed the following three methods: change-score analysis, change-score analysis using GAMs, and change-score analysis using GAMs without considering the EEG data obtained before stress (cardiac catheterization).

### Change-score analysis

Table 3 shows the 84 patients who were analyzed using the change-score analysis. Six multiple regression parameters were evaluated. The pre-alpha activity, pre-meanNN, and difference-meanNN were the three main predictive factors used to recognize relaxing states on the basis of difference-alpha activity. Moreover, the pre-test alpha activity could influence the occurrence of difference-alpha activity because of its direct effect on the difference-meanNN and its indirect effect on pre-meanNN. Furthermore, the age, pre-log-Slopes 1–5, and pre-Slopes 1–5 were minor predictive factors for the difference-alpha activity. The results of the six regression parameters shed light on the mechanism of the major predictive factors of the difference-alpha activity, and the multiple R-squared value was 0.6509. This proposed method achieved an overall accuracy of 80.7% in recognizing changes in the EEG alpha waves.

### Change-score analysis using generalized additive models

The GAMs were used to detect the nonlinear effects of continuous covariates and identify appropriate cutoff points for discretizing a continuous covariate. GAMs enable the direct observation of the partial effect of a continuous covariance; therefore, the cutoff points can be easily selected. Supplementary Table S3 shows the continuous ECG parameters that were analyzed using the GAMs. The GAM of all patients is shown in supplementary Figs S1 to S19.

After stepwise variable selection, five GAM variables were included in the change-score analysis, and the multiple R-squared value increased to 0.7485, as shown in Table 4. The pre-alpha activity and difference-meanNN were the predominant predictive factors of difference-alpha activity. Furthermore, the difference-meanNN, difference-the ratio of LF over HF and GAM’s HF were minor x values for difference-alpha activity. The proposed method achieved an overall accuracy of 86.5% in recognizing changes in EEG alpha waves.

### Change-score analysis using generalized additive models without electroencephalography data obtained before stress (cardiac catheterization)

This model was developed for wearable sensor nodes that, based on change-score analysis, identified linear and nonlinear indices of HRV, hypertension, and neurotransmitter levels without considering the electroencephalography data obtained before stress. As shown in Table 5, 12 multiple regression parameters were evaluated. Slopes 1–5 were the main predictive factors of the difference-alpha activity. In contrast to the change-score analysis and the change-score analysis using GAMs, nonlinear analyses are useful for predicting the difference-alpha activity. Furthermore, the difference-meanNN and difference-the ratio of LF over HF were minor predictive factors for the difference-alpha activity. In addition, the serum neurotransmitter levels, hypertension, and serotonin were included in the model. After stepwise variable selection, the multiple R-squared value was 0.4988. The proposed method achieved an overall accuracy of 70.6% in recognizing changes in EEG alpha waves. Although the prediction accuracy was relatively low, continuous EEG parameters need not be considered. Hence, HRV analyses are sufficient for assessing the autonomic function.

## Discussion

In movement control, the brain commands the motor system to produce the desired movements. The brain appears to control the motor system, but the afferent input from the motor system may strengthen or weaken the synaptic connections, thereby reorganizing the brain and leading to functional and structural changes in the brain. We posited that the brain interacts similarly with the heart. In our previous study^17^, we demonstrated that there are correlations between the signal complexity of cardiac and cerebral activities. In the present study, we used cardiac catheterization as a stress event to evaluate the predictive power of post-stress brain activity by considering the ECG complexity parameters. As mentioned previously, EEG alpha waves represent a relaxing index^28^. Therefore, we developed three methods, which primarily involve ECG and EEG parameters, for assessing the human relaxing state after stress.

A numerical study of estimating the human relaxing state was developed in the literature^4^. The present method requires purely parametric values, and the human relaxing state can be calculated dynamically. This method mainly requires HRV metrics and the instantaneous power spectrum of EEG during the estimated relaxing states. Because this method was further incorporated with clinical data, such as the personal profile, diagnosis or serum neurotransmitter, it should be able to accurately characterize the relaxation response.

Two nonlinear methods, MSE and DFA, were used to quantify the heartbeat complexity and variability of heartbeat time series to provide information about the autonomic function. Because the connection between brain and cardiac activity has been demonstrated to be highly complex, HRV is a conventional metric for the pathophysiology and psychopathology^29^. Although conventional HRV metrics are reliable for predicting human relaxing states, the prediction accuracy is limited in the presence of frequent arrhythmias^30^. In this study, MSE and DFA were used to derive nonstationary and nonlinear dynamic changes from the heart rate time series^17^,^31^,^32^. The short-fitted slope (Slopes 1–5) of the curve obtained using MSE was considered when the change-score analysis was used in the change-score analysis. Moreover, when we used generalized additive models to increase the prediction accuracy, the short-fitted area (Areas 1–5) and long-fitted area (Areas 6–20) were used. Compared with conventional linear HRV indices, the nonlinear indices of the employed method had higher prediction ability for cardiac catheterization.

Previous studies have indicated that alpha activity reflects the selective cortical inhibition that is involved in global neural integration^33^,^34^. This feature of alpha activity has received considerable attention in cognitive neuroscience. The cognitive literature suggests that alpha activity is potentially involved in various other physiological processes, such as relaxing, or in specific regions of the brain^35^. Therefore, we hypothesized that alpha power is correlated with the relaxing state because the effect of neural inhibition could change emotions according to the physiological and pathological states. Therefore, short-time Fourier transform was used to quantify the spectral power of alpha activity from a 4-min time series of alpha activity. Our results showed that when the post EEG alpha waves were considered in the change-score analysis, the predictive accuracy increased from 70.6% to 80.7%.

Although we employed change-score analysis to estimate the human relaxing state after stress, numerous nonlinear HRV metrics (listed in Supplementary Table 3) were not considered. Consequently, the generalized additive models were fit to detect the nonlinear effects of continuous covariates^36^. In this study, generalized additive models were used to visualize the relationship of nonlinear HRV metrics with the difference between the pre- and posttest alpha wave responses. On the basis of graphical visualization, nonlinear HRV metrics can be used to identify the appropriate cutoff points for discretizing a continuous covariate (see Supplementary Fig. S1–S19). Our results for the ratio between the LF and HF and for the HF were considered in the prediction methods after fitting the generalized additive models. A comparison between the proposed prediction model and the proposed prediction model, without the generalized additive models, showed that the time-domain HRV metrics were the only parameters that could be considered to predict human relaxing states.

In this study, an innovative aspect of the method is the use of HRV and EEG to fit the regression model and the observed data for effect estimation and outcome prediction. Three methods were used for assessing the difference between the pre- and post-test alpha wave response. A change-score analysis model identified six mathematical parameters in the HRV and EEG that may facilitate predicting post-stress alpha waves. Conventional linear HRV analysis involves two metrics, the meanNN (pre-) and meanNN (difference), which provide positive and considerably low estimates. In addition, MSE analysis involves two metrics, Slopes 1–5 (pre-) and Log-areas 1–5 (pre-). Among the clinical parameters, only age was associated with high predictability. Furthermore, the change-score analysis model using GAMs identified 11 regression metrics, and five GAM variables were included in the predictive model. All GAM estimated values of the HRV analysis were positive at specific cutoff points. In addition, five predictive parameters that had been identified in previous studies were retained. Considering the practical application on wearable sensor nodes^11^, the EEG data obtained before stress (cardiac catheterization) were not used in the change-score analysis. Five regression metrics are used in conventional linear HRV analysis. In the present study, sdNN(pre-), pNN20 (pre-), and LF/HF (difference) were new metrics that were added to the predictive model. The variables associated with clinical parameters included hypertension. An estimated value of serotonin was, for the first time, considered personalized in change-score analysis. Drugs that alter the serotonin levels are used to treat depression and generalized anxiety disorder. Therefore, it is reasonable for serotonin to be included in this model. This model shows that when we did not include the prestress alpha activity, the difference between the pre- and posttest alpha wave responses require more parameters to estimate the relaxing states. Of note, three predictable models did not include the treatment category of clinical data, which means that our proposed method can be applied to healthy subjects.

This study had numerous limitations. First, there was no control group in the entire relaxing condition, such in a home setting. All study subjects were patients with a relative anxiety status. Therefore, another study is needed to investigate the relationship between the brain and heart in subjects with a relaxing condition. Second, all ECG and EEG data were recorded in normal “free running” conditions, which implied the possible existence of interfering factors (e.g., physical activities and different relaxing states). Third, the database was based on data collected for a period of 1 h. Hence, dynamic noise or nonstationary artifacts might have affected the signal properties. Although the segments of ECC and EEG were detrended using a deterministic nonlinear method (empirical mode decomposition) discussed in previous studies^37^, we did not assess the noise features or noise level to obtain more detailed information. Finally, some parameters related to the inhibitory characteristics reflected by EEG, such as cortical inhibitory mechanisms, were not considered in this study^38^. Therefore, it is necessary to develop a specific mathematical algorithm for quantifying global neural integration.

In conclusion, the method of predictive analysis demonstrates the potential of the interrelations between heartbeat and neural communication networks by using an accurate quantification equation involving change-score analysis, which is lacking in most previous studies. Accordingly, to increase the probabilistic accuracy, we developed a method to test our hypothesis. In this method, although conventional HRV analysis enables the quantification of a heartbeat, nonlinear methods provide additional information about the heartbeat complexity and fractal correlation. The proposed method achieved an overall accuracy of 80.7 and 86.5% in recognizing alpha-wave changes after stress basis on the change-score analysis and GAMs, respectively. Therefore, the complexity of cardiac electrical signals could be used to predict changes in the EEG alpha waves after stress.

## Methods

The ethics committee on human research of National Taiwan University Hospital (NTUH) approved the study (IRB-201206070RIC). All participants or their surrogates gave written informed consent. The investigation conformed to the principles of the Declaration of Helsinki.

### Data collection

Patients with angina and positive stress tests who had undergone cardiac catheterization uneventfully were enrolled in this study from August 2012 to May 2014. One day before and immediately after cardiac catheterization, these patients underwent 1-hour EEG (Neuron-Spectrum-3, Neurosoft Company; Digital Neurophysiological Systems, Russia) monitoring with a sampling rate of 512 Hz and resolution of 16 bits. Continuous ECG recordings were extracted from the resting awake EEG for each patient. The RR interval (RRI) of ECG was determined using an automated detection algorithm, and the annotated file was carefully inspected and corrected for the extraction of the RRIs. Surface EEG was conducted using 19 electrodes of the international standard 10–20 system (FP2, F3, F4, FZ, C3, C4, CZ, P3, P4, PZ, O1, O2, F7, F8, T3, T4, T5, and T6). All patient data, including artifacts, such as eye movements, blinks, and muscle activities, were saved in text files for offline analysis on a laptop, and 4-min segments of the time-series were chosen from each file. Medical history, including demography and medication, was carefully recorded. Blood was sampled during cardiac catheterization.

### Time- and frequency-based analysis

Conventional HRV metrics were calculated in the time and frequency domains according to the guidelines developed by the Task Force of the European Society of Cardiology^12^.

The patients were diagnosed using metrics in the time and frequency domains. The time domain metrics were as follows: the mean value of RRI time series (meanNN), standard deviation of the RRI time series (sdNN), root mean square of the differences between successive RRIs (rMSSD), percentage of absolute differences greater than 20 ms in normal RRIs (pNN20), and percentage of absolute differences greater than 50 ms in normal RRIs (pNN50). The frequency domain metrics were as follows: high-frequency component (HF, 0.15–0.4 Hz), low-frequency component (LF, 0.04–0.15 Hz), and the ratio between the low-frequency and high-frequency components (LF/HF), which were computed according to the average power spectrum.

### Multiscale entropy analysis

MSE analysis can be used to estimate the complex pattern of a time series and evaluate the complexity of physiological signals on multiple time scales^21^.

The MSE analysis method consists of two main steps: coarse-graining of the signals into different time scales and quantifying the degree of irregularity in each coarse-grained time series using sample entropy (SpEn)^39^. Coarse-graining yields a one-dimensional discrete time series {{*x*_1_,…, *x*_*i*_,…, *x*_N_}. In addition, the consecutive coarse-grained time series, {*y*^*τ*^}, which corresponds to the scale factor *τ*, can be constructed. To construct a coarse-grained series, the original time series should first be divided into *N*/*τ* nonoverlapping windows of length *τ*. Subsequently, the data points within each window should be averaged. Generally, each element of the coarse-grained series is calculated as follows:


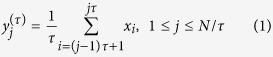


With a scale factor of one, which corresponds to *τ* = 1, the coarse-grained series 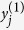 is exactly the same as the original one.

In addition, the MSE curve can be divided into long- and short-term curves. The different complexities of the scales can be useful for clinical categorization and can be divided into four types of parameters: (a) when only slope is considered, the first 5 scales are defined as the short-fitted slope (Slopes 1–5), and the final 15 scales are defined as the long-fitted slope (Slopes 6–20); and (b) when only the area is considered, the first 5 scales are defined as a short-fitted area (Areas 1–5), and the final 15 scales are defined as a long-fitted area (Areas 6–20). Although MSE analysis has been applied to physiological signals, the entropy values are sensitive to very-low-frequency noise and nonstationary artifacts, especially trends. Accordingly, the de-trending process should be performed prior to MSE analysis^37^. In the present study, the ensemble empirical mode decomposition method was adopted to remove the trend of the RRI signals.

### Detrended fluctuation analysis

DFA can be used to quantify nonstationary and nonlinear dynamic changes in the heart rate time series. Recently, DFA has been widely used to analyze cardiac problems by inputting patients’ RRI signals^40^. To calculate the scaling exponents in DFA, a given time series *x*(*i*), 1 ≤ i ≤ *N,* which should first be integrated as follows:


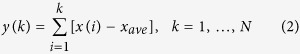


where *x*_*ave*_ is the mean of time series *x*(*i*). Subsequently, the integrated time series is divided into boxes of equal length n. Each box contains a least square line for fitting the divided time series, and the time series can be detrended by subtracting the local trend. The root-mean-square fluctuation of the integrated time series can be obtained as follows:


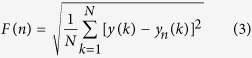


where the average fluctuations *F*(*n*) are represented as a function of the box size. The average fluctuations of the data sets indicate whether the crossover phenomenon exists between short- and long-term scales.

The short-term (<11 beats, alpha_1) and long-term (>11 beats, alpha_2) fractal correlation exponents were calculated to obtain a clearer understanding of the fractal correlation property of the biological system^23^. In addition, the heartbeat dynamics were characterized by a scaling exponent ∝, and the slope of the linear relationship was estimated according to the log-log plot of fluctuations versus box sizes.

### Electroencephalography analysis using short-time Fourier Transform

**Figure 2:**
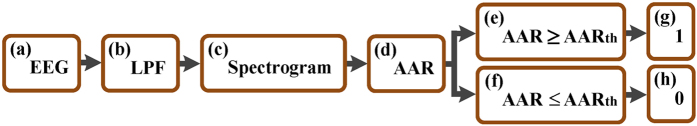
STFT along with a spectrogram and fixed window size can be used for localizing signals. *w*(*t*) and *x*(*t*) denote the window and EEG signal, respectively, and the EEG signals are expressed in the time–frequency domain.

In STFT, a short time window is applied to biomedical signals^41^,^42^ and a series of Fourier transforms are performed within this window as the window slides across all data; STFT provides a time-frequency representation of the biomedical signal (Fig. 2). STFT can provide an instantaneous estimate of the time-varying energy because the Fourier transform can be adapted for analyzing a localized signal. Mathematically, STFT is defined as follows:


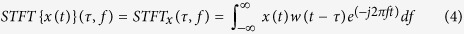


where *w*(*t*) is a symmetric window function and *x*(*t*) is the signal to be transformed. *STFT*_*x*_(*τ*, *f*) uses a complex exponential basis function as the basis. The squared magnitude of STFT is referred to as a spectrogram:


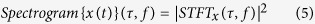


where *τ* is discrete and *f* is continuous.

All analyses were performed using MATLAB (The MathWorks, Natick, MA, USA). The data were low-pass filtered with a cutoff frequency of 55 Hz to remove power line noise, and the power changes in the EEG following movement execution were estimated for all patients using a fast Fourier transform with windows of 1024 samples, a Hanning window with a width of 0.5 s, and a 70% overlap until all the EEG signals were analyzed.

The objective of using STFT is to assess the relaxing state of a subject exclusively through EEG analysis. Hence, the alpha activity is an essential metric for assessing relaxed wakefulness when visual processes are engaged by opening the eyes^43^. In the present study, we calculated only the alpha-activity-ratio related EEG signals using time–frequency analysis. The alpha-activity-ratio (AAR) obtained through EEG was investigated by comparing the alpha band (8–12 Hz) to the full-band EEG (1–55 Hz). Mathematically, the AAR is defined as follows:


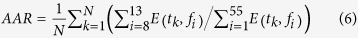


where *E*(*t*_*k*_, *f*_*i*_) is the spectrum of the *i*-th bin in STFT, *t*_*k*_ denotes the time indices, and N is the total time length. On the basis of the obtained AARs, a significant level (*AAR*_*th*_) can be identified to quantify the intensity of the alpha waves.

**Figure 3:**
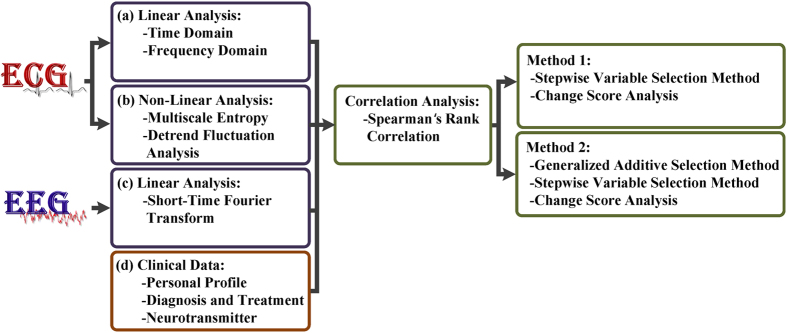
Functional alpha activity computation flowchart. (**a**) The input EEG signals were obtained from 19 electrodes of the international standard 10–20 systems. (**b**) The data were passed through a low-pass filter (LPF) with a cutoff frequency of 55 Hz. (**c**) STFT is presented as a spectrogram. (**d**) The AAR was investigated by comparing the alpha band (8–12 Hz) with the full-band EEG (1–55 Hz). (**e**,**f**) AAR_th_ is a threshold and (**g**,**h**) denotes the energy change at a significant level for all channels.

In this study, we proposed a framework for calculating significant changes in the average time–frequency power density. This method consists of the following steps: (a) calculating the time–frequency power density, (b) dividing the time–frequency plane in which the alpha wave power density is calculated, (c) selecting a threshold for all channels, and (d) determining the energy change at the significant level for all channels. The process flow block diagram is presented in Fig. 3.

### Serum neurotransmitter measurement

The levels of serum neurotransmitters, including orphanin FQ, dopamine, and serotonin, were measured. The orphanin FQ level was measured using an ELISA Kit (MyBioSource, San Diego, CA, USA), the serotonin level was measured using a serotonin ELISA Kit (Abcam, Cambridge, United Kingdom), and the dopamine level was measured using high-performance liquid chromatography.

### Statistical analysis

Statistical analysis was performed using the R 3.1.1 software (R Foundation for Statistical Computing, Vienna, Austria). A two-sided *p* value less than 0.05 was considered statistically significant. Data were expressed as the mean ± standard deviation. Differences in the serum neurotransmitter levels between the groups of patients were assessed using the Mann-Whitney U Test. Continuous data were assessed using the Mann-Whitney U Test, and categorical data were assessed using Fisher’s exact test. The statistical analysis consisted of Spearman’s rank correlation, a stepwise variable selection method, and change-score analysis.

The objective of conducting a regression analysis of the change scores (i.e., the difference between the pre- and posttest response) was to determine one or a few parsimonious regression models that fit the observed data well for effect estimation and outcome prediction. To ensure that the analysis quality was high, basic model-fitting techniques for variable selection, change-score analysis of pre- and posttest data, and regression diagnostics and remedies (e.g., ensuring that multicollinearity was not present and detecting influential cases) were applied. Before model fitting, the continuous ECG and EEG parameters were analyzed using Spearman’s rank correlation to remove confounding variables. The significance level for the confounding effects was set to be greater than 0.8.

The stepwise variable selection procedure (with iterations between the forward and backward steps) was used to identify the optimal candidate final regression model. All relevant univariate significant and nonsignificant covariates (listed in Tables 1 and 2) and some of their interactions were included in the variable list to be selected. The significance level for entry and stay was conservatively set to 0.15. Subsequently, the optimal candidate final regression model was manually identified by dropping the covariates with *p* values greater than 0.05, one at a time, until all regression coefficients were significantly different from zero.

The change-score analysis was used for changing subject *i* by Δ*Y*_*i*_ = *Y*_*i*1_ − *Y*_*i*0_, where *Y*_*i*0_ and *Y*_*i*1_ are the measured continuous pre- and posttest response variables, respectively^44^. Furthermore, the regression analysis of the mean change between two groups can be defined as follows:


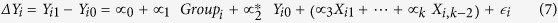


where Group = 0 denotes the control group and Group = 1 denotes the treatment group; mathematically, 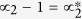. *X*_*i*1_, *X*_*i*2_, ..., *X*_*ik*_ are the other covariates that can affect the mean of the continuous response variable and 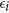 is the random error. As a general rule, when Δ*Y*_*i*_ = *Y*_*i*1_ − *Y*_*i*0_ is specified as the response variable, the baseline response *Y*_*i*0_ must be introduced on the right-hand side of the regression equation.

The *R*^2^ statistic (0 ≤ *R*^2^ ≤ 1) for a linear regression model represents the correlation between the observed and predicted response values and indicates how much of the response variability is explained by the covariates in the linear regression model.

Finally, generalized additive models (GAMs)^45^ were used to increase the prediction accuracy. The GAMs were fit to detect the nonlinear effects of continuous covariates and identify appropriate cutoff points for discretizing a continuous covariate. Thus, we obtained unbiased estimates of the covariates’ effects and more accurate response predictions.

## Additional Information

**How to cite this article**: Chiu, H.-C. *et al.* Complexity of cardiac signals for predicting changes in alpha-waves after stress in patients undergoing cardiac catheterization. *Sci. Rep.* **5**, 13315; doi: 10.1038/srep13315 (2015).

## Supplementary Material

Supplementary Information

## Figures and Tables

**Table 1:** Demographic data of the patients.

	Total patients(n=84)	Revascularization treatment		
		**Without revascularization treatment (n = 19)**	**With revascularization treatment (n = 65)**	
Male/female	70/14	12/7	58/7	P = 0.0133
Age	64.2 ± 11.9	62.6 ± 10.7	64.6 ± 12.3	P = 0.5634
Body mass index	26.8 ± 3.6	28.2 ± 3.6	26.3 ± 3.50	P = 0.0563
Estimated glomerular filtration rate	1(24)/0(60)	1(19)/0(46)	1(5)/0(14)	P = 1
Fasting glucose	135 ± 37	167 ± 51	131 ± 34	P = 0.1088
Triglyceride	166 ± 88	168 ± 90	165 ± 88	P = 0.9531
Total cholesterol	167 ± 38	173 ± 28	166 ± 41	P = 0.1473
Uric acid	8.7 ± 15.5	9.4 ± 11.0	8.5 ± 16.7	P = 0.1472
Mean arterial blood pressure (before cardiac catheterization)	96.4 ± 12.2	93.7 ± 11.3	97.2 ± 12.5	P = 0.3522
Mean arterial blood pressure (after cardiac catheterization)	92.5 ± 11.8	92.1 ± 9.6	92.6 ± 12.4	P = 0.8937
Hypertension	1(75)/0(9)	1(17)/0(2)	1(58)/0(7)	P = 1
Diabetes mellitus	1(34)/0(50)	1(4)/0(15)	1(30)/0(35)	P = 0.06447
High cholesterol	1(66)/0(18)	1(13)/0(6)	1(53)/0(12)	P = 0.2228
Current smoker	1(49)/0(35)	1(10)/0(9)	1(39)/0(26)	P = 0.6046
Heart failure	1(12)/0(72)	1(3)/0(16)	1(9)/0(56)	P = 1
Peripheral arterial occlusive disease	1(2)/0(82)	1(0)/0(19)	1(2)/0(63)	P = 1

**Table 2:** Effect of the revascularization treatment on the autonomic activities, brain waves and serum neurotransmitter.

	Total patients(n=84)	Revascularization treatment		
		**Without revascularization treatment (n = 19)**	**With revascularization treatment (n = 65)**	
Serum neurotransmitter				
Dopamine	212.9 ± 220.8	148.96 ± 201.66	231.60 ± 224.09	P = 0.0470
Orphanin-FQ	86.7 ± 75.8	77.51 ± 80.45	89.44 ± 74.77	P = 0.7003
Serotonin	176.8 ± 140.8	153.76 ± 150.69	183.56 ± 138.26	P = 0.2049
EEG				
Alpha activity (pre-)	12.8 ± 6.1	13.3 ± 6.2	12.7 ± 6.2	P = 0.6629
Alpha activity (post-)	14.8 ± 4.9	16.7 ± 2.7	14.2 ± 5.4	P = 0.2321
Alpha activity (Difference-)	1.9 ± 5.5	3.4 ± 5.2	1.6 ± 5.5	P = 0.2870
ECG (Linear variable)				
meanNN (pre-)	910.5 ± 141.1	954.3 ± 127.6	897.7 ± 143.2	P = 0.0932
meanNN (post-)	903.0 ± 147.2	942.1 ± 102.3	891.6 ± 156.8	P = 0.1290
Log-meanNN (pre-)	6.8 ± 0.2	6.9 ± 0.1	6.8 ± 0.2	P = 0.0932
Log-meanNN (post-)	6.7 ± 0.2	6.8 ± 0.1	6.8 ± 0.2	P = 0.1290
sdNN (pre-)	97.1 ± 63.8	118.8 ± 69.8	90.8 ± 61.0	P = 0.0871
sdNN (post-)	98.8 ± 66.8	115.1 ± 62.2	94.1 ± 67.8	P = 0.0708
Log-sdNN (pre-)	4.4 ± 0.6	4.6 ± 0.7	4.3 ± 0.6	P = 0.0871
Log-sdNN (post-)	4.4 ± 0.5	4.6 ± 0.5	4.3 ± 0.6	P = 0.0708
pNN20 (pre-)	0.3885 ± 0.2393	0.4974 ± 0.2553	0.3567 ± 0.2267	P = 0.0262
pNN20 (post-)	0.3829 ± 0.2550	0.5 ± 0.2	0.4 ± 0.3	P = 0.0708
Log-pNN20 (pre-)	−1.19 ± 0.77	−0.87 ± 0.68	−1.27 ± 0.77	P = 0.0262
Log-pNN20 (post-)	−1.30 ± 0.98	−0.9 ± 0.6	−1.4 ± 1.0	P = 0.0708
pNN50 (pre-)	0.1619 ± 0.2271	0.2414 ± 0.2911	0.1387 ± 0.2016	P = 0.1111
pNN50 (post-)	0.1689 ± 0.2286	0.2158 ± 0.2442	0.1552 ± 0.2239	P = 0.0912
Log-pNN50 (pre-)	−2.78 ± 1.52	−2.39 ± 1.83	−2.89 ± 1.41	P = 0.1111
Log-pNN50 (post-)	−2.71 ± 1.52	−2.20 ± 1.29	−2.86 ± 1.55	P = 0.0912
rMMSD (pre-)	111.3 ± 99.9	136.4 ± 109.3	104.0 ± 96.7	P = 0.2755
rMMSD (post-)	113.8 ± 103.6	126.5 ± 91.0	110.1 ± 107.4	P = 0.3359
Log-rMMSD (pre-)	4.4 ± 0.8	4.6 ± 0.9	4.4 ± 0.7	P = 0.2755
Log-rMMSD (post-)	4.4 ± 0.8	4.6 ± 0.8	4.4 ± 0.8	P = 0.3386
LF (pre-)	2158.9 ± 6160.9	4465.3 ± 11862.0	1484.7 ± 2758.8	P = 0.2311
LF (post-)	6704.0 ± 10244.4	11857.8 ± 15274.0	5197.5 ± 7765.2	P = 0.0517
Log-LF (pre-)	6.4 ± 1.5	6.8 ± 1.8	6.3 ± 1.4	P = 0.2311
Log-LF (post-)	8.0 ± 1.3	8.6 ± 1.4	7.8 ± 1.3	P = 0.0517
HF (pre-)	4112.9 ± 15952.1	6344.1 ± 18366.6	3460.8 ± 15271.9	P = 0.4542
HF (post-)	8774.7 ± 14684.1	13801.1 ± 20361.2	7305.4 ± 12383.2	P = 0.2755
Log-HF (pre-)	6.5 ± 1.7	6.8 ± 2.1	6.4 ± 1.6	P = 0.4542
Log-HF (post-)	8.0 ± 1.5	8.5 ± 1.6	7.9 ± 1.5	P = 0.2755
LF/HF (pre-)	1.0 ± 0.5	1.1 ± 0.5	1.0 ± 0.5	P = 0.5212
LF/HF (post-)	1.1 ± 0.6	1.3 ± 0.8	1.0 ± 0.5	P = 0.2616
Log-LF/HF (pre-)	−0.1127 ± 0.5095	−0.0133 ± 0.4433	−0.1416 ± 0.5268	P = 0.5212
Log-LF/HF (post-)	−0.0402 ± 0.5222	0.1148 ± 0.5398	−0.0856 ± 0.5123	P = 0.2616
meanNN (Difference-)	−7.4 ± 94.73	−12.2 ± 62.4	−6.1 ± 102.6	P = 0.7322
Log-meanNN (Difference-)	−0.01 ± 0.10	−0.01 ± 0.06	−0.01 ± 0.11	P = 0.5782
sdNN (Difference-)	1.73 ± 45.1	−3.7 ± 59.9	3.3 ± 40.2	P = 0.7810
Log-sdNN (Difference-)	0.011 ± 0.432	0.026 ± 0.474	0.008 ± 0.416	P = 0.9318
pNN20 (Difference-)	−0.01 ± 0.17	−0.021 ± 0.149	0.001 ± 0.181	P = 0.6767
Log-pNN20 (Difference-)	−0.12 ± 0.63	−0.03 ± 0.38	−0.14 ± 0.69	P = 0.5212
pNN50 (Difference-)	0.01 ± 0.16	−0.02 ± 0.19	0.02 ± 0.15	P = 0.5782
Log-pNN50 (Difference-)	0.07 ± 1.06	0.19 ± 1.14	0.03 ± 1.04	P = 0.3924
rMMSD (Difference-)	2.4 ± 64.3	−9.9 ± 91.0	6.1 ± 54.6	P = 0.6457
Log-rMMSD (Difference-)	0.0036 ± 0.5814	0.0142 ± 0.6444	0.0005 ± 0.5671	P = 0.7322
LF (Difference-)	4545.1 ± 11820.4	7392.5 ± 20403.7	3712.8 ± 7810.5	P = 0.0932
Log-LF (Difference-)	1.6 ± 1.7	1.8 ± 1.9	1.5 ± 1.6	P = 0.2802
HF (Difference-)	4661.7 ± 21113.67	7457.0 ± 28693.2	3844.7 ± 18535.9	P = 0.3253
Log-HF (Difference-)	1.5 ± 1.8	1.7 ± 2.0	1.5 ± 1.7	P = 0.6380
LF/HF (Difference-)	0.09 ± 0.52	0.21 ± 0.63	0.06 ± 0.48	P = 0.0891
Log-LF/HF (Difference-)	0.07 ± 0.43	0.12 ± 0.45	0.06 ± 0.42	P = 0.1415
ECG (Nonlinear variable)				
Slopes 1–5 (pre-)	0.0029 ± 0.0713	−0.0122 ± 0.0843	0.0073 ± 0.067	P = 0.4738
Slopes 1–5 (post-)	0.0037 ± 0.0819	0.0253 ± 0.0970	−0.0025 ± 0.0767	P = 0.2898
Slopes 6–20 (pre-)	0.0049 ± 0.0166	−0.0011 ± 0.0179	0.0067 ± 0.0159	P = 0.1136
Slopes 6–20 (post-)	0.0096 ± 0.0173	0.0060 ± 0.0187	0.0106 ± 0.0169	P = 0.2947
Area 1–5 (pre-)	4.07 ± 1.44	4.47 ± 1.72	3.95 ± 1.34	P = 0.3359
Area 1–5 (post-)	3.94 ± 1.52	4.28 ± 1.89	3.84 ± 1.39	P = 0.2616
Log_Area 1–5 (pre-)	3.1 ± 4.9	2.58 ± 3.43	3.3 ± 5.2	P = 0.4607
Log_Area 1–5 (post-)	3.1 ± 5.1	2.8 ± 4.6	3.2 ± 5.2	P = 0.3749
Area 6–20 (pre-)	14.7 ± 6.8	15.7 ± 6.5	14.4 ± 6.9	P = 0.4938
Area 6–20 (post-)	14.9 ± 7.2	16.3 ± 7.8	14.5 ± 7.1	P = 0.1995
Log_Area 6–20 (pre-)	2.8 ± 0.3	2.8 ± 0.3	2.7 ± 0.4	P = 0.7892
Log_Area 6–20 (post-)	2.7 ± 0.4	2.8 ± 0.6	2.7 ± 0.4	P = 0.2149
∝_1_ (pre-)	0.75 ± 0.19	0.78 ± 0.21	0.74 ± 0.18	P = 0.5422
∝_1_ (post-)	0.78 ± 0.21	0.83 ± 0.22	0.76 ± 0.20	P = 0.2708
Log_∝_1_ (pre-)	−0.32 ± 0.29	−0.28 ± 0.29	−0.33 ± 0.30	P = 0.5422
Log_∝_1_ (post-)	−0.29 ± 0.32	−0.21 ± 0.28	−0.32 ± 0.33	P = 0.2708
∝_2_ (pre-)	0.78 ± 0.13	0.78 ± 0.14	0.77 ± 0.13	P = 0.6611
∝_2_ (post-)	0.75 ± 0.12	0.72 ± 0.13	0.75 ± 0.12	P = 0.2898
Log_∝_2_ (pre-)	−0.27 ± 0.16	−0.26 ± 0.18	−0.27 ± 0.16	P = 0.6611
Log_∝_2_ (post-)	−0.31 ± 0.17	−0.34 ± 0.17	0.29 ± 0.17	P = 0.2898
Slopes 1–5 (Difference-)	0.0008 ± 0.0776	0.0375 ± 0.0878	−0.0098 ± 0.0716	P = 0.0833
Slopes 6–20 (Difference-)	0.0046 ± 0.0168	0.0072 ± 0.0171	0.0038 ± 0.0168	P = 0.6611
Areas 1–5 (Difference-)	−0.13 ± 1.25	−0.19 ± 1.13	−0.11 ± 1.29	P = 0.8979
Log_Areas 1–5 (Difference-)	−0.0044 ± 1.2235	0.2118 ± 1.4369	−0.0676 ± 1.1587	P = 0.6845
Areas 6–20 (Difference-)	0.2 ± 4.7	0.6 ± 4.2	0.1 ± 4.9	P = 0.8223
Log_Areas 6–20 (Difference-)	−0.01 ± 0.41	−0.008 ± 0.418	−0.009 ± 0.414	P = 0.6304
∝_1_ (Difference-)	0.02 ± 0.17	0.05 ± 0.14	0.02 ± 0.18	P = 0.2997
Log-∝_1_ (Difference-)	0.03 ± 0.25	0.071 ± 0.175	0.013 ± 0.263	P = 0.3253
∝_2_ (Difference-)	−0.03 ± 0.13	−0.06 ± 0.13	−0.02 ± 0.13	P = 0.2482
Log- ∝_2_ (Difference-)	−0.04 ± 0.18	10.074 ± 0.175	−0.030 ± 0.179	P = 0.3306

**Table 3:** Multiple regression analysis of the difference-alpha activity.

Parameter	**Total patients (N** **=** **84)**			
	**Estimate**	**Std. Error**	**t value**	**p-value**
(Intercept)	3.2361	3.0405	1.064	p = 0.2905
meanNN (Pre-)	0.0124	0.0029	4.350	p < 0.0001
Slope1_5 (Pre-)	−12.6317	5.4399	−2.322	p = 0.0229
Log_Area 1_5 (Pre-)	0.1948	0.0760	2.562	p = 0.0124
Alpha_activity (Pre-)	−0.5329	0.0611	−8.717	p < 0.0001
meanNN (Difference)	0.0201	0.0043	4.688	p < 0.0001
Age	−0.0963	0.0329	−2.931	p = 0.0045

Residual standard error: 3.3572 on 77 degrees of freedom.

Multiple R-squared: 0.6509, Adjusted R-squared: 0.6237.

F-statistic: 23.9266 on 6 and 77 DF, p-value < 0.0001.

**Table 4:** Multiple regression analysis of the difference-alpha activity using GAMs.

Parameter	**Total patients (N** **=** **84)**			
	**Estimate**	**Std. Error**	**t value**	**p-value**
(Intercept)	0.9668	2.7122	0.356	p = 0.7225
meanNN (Pre-)	0.0104	0.0026	3.992	p = 0.0002
Slope1_5 (Pre-)	−15.7228	4.9149	−3.199	p = 0.0021
Alpha_activity (Pre-)	−0.5704	0.0555	−10.278	p< 0.0001
meanNN (Difference)	0.0219	0.0039	5.597	p< 0.0001
LF_HF (Difference)	−2.8915	0.7175	−4.030	p = 0.0001
0.017 ≤pNN50(Pre-) ≤ 0.176	1.9557	0.6820	2.867	p = 0.0054
1.229 ≤LF/HF (Pre-) ≤1.98	1.9867	0.8599	2.310	p = 0.0237
2.183 ≤ Log -Area(Pre-) ≤ 2.895	2.0038	0.7134	2.809	p = 0.0064
−0.164 ≤ pNN20 (Difference) ≤ 0.007	1.5934	0.6858	2.323	p = 0.0230
HF (Difference) ≤ 376.933 and HF (Difference) ≥ 25327.388	2.5186	0.6999	3.598	p = 0.0006
Age	−0.0776	0.0309	−2.506	p = 0.0145

Residual standard error: 2.9466 on 72 degrees of freedom.

Multiple R-squared: 0.7485, Adjusted R-squared: 0.7101.

F-statistic: 19.4842 on 11 and 72 DF, p-value: 0.

**Table 5:** Change-score analysis performed using GAMs without considering the pre-alpha activity.

Parameter	**Total patients (N** **=** **84)**			
	**Estimate**	**Std. Error**	**t value**	**p-value**
(Intercept)	−7.3293	3.5819	−2.046	p = 0.0444
meanNN (Pre-)	0.0091	0.0037	2.449	p = 0.0170
sdNN (Pre-)	0.0369	0.0168	2.193	p = 0.0326
pNN20 (Pre-)	−9.8173	4.4838	−2.190	p = 0.0320
Slope1_5 (Pre-)	−36.8229	9.0475	−4.070	p = 0.0001
meanNN (Difference)	0.0176	0.0057	3.102	p = 0.0028
LF/HF (Difference)	−2.7241	0.9346	−2.915	p = 0.0048
3.81 ≤ Log_rMSSD (Pre-) ≤ 5.348	2.6421	1.0521	2.511	p = 0.0143
1.229 ≤ LF/HF (Pre-) ≤ 1.98	2.6666	1.2603	2.116	p = 0.0379
Log_Area 1~5 (Pre-) ≤ 1.007	−4.2005	1.7325	−2.425	p = 0.0179
HF (Difference) ≤ 376.933 and HF (Difference) ≥ 25327.388	2.7330	1.08173	2.526	p = 0.0138
Hypertension	−3.5953	1.3556	−2.652	p = 0.0099
Log-Serotonin	0.5052	0.2083	2.426	p = 0.0178

Residual standard error: 4.1893 on 71 degrees of freedom.

Multiple R-squared: 0.4988, Adjusted R-squared: 0.414.

F-statistic: 5.8875 on 12 and 71 DF, p-value: 0.

## References

[b1] Benarroch E. E. The Central Autonomic Network: Functional Organization, Dysfunction, and Perspective. Mayo Clinic Proceedings 68, 988–1001 (1993). 8412366 10.1016/s0025-6196(12)62272-1

[b2] Kreibig S. D. Autonomic nervous system activity in emotion: A review. Biological Psychology 84, 394–421 (2010). 20371374 10.1016/j.biopsycho.2010.03.010

[b3] Ekman P., Levenson R. W. & Friesen W. V. Autonomiv Nervous-Systrm Activity Distinguishes Among Emotions. Science 221, 1208–1210 (1983). 6612338 10.1126/science.6612338

[b4] Calvo R. A. & D’Mello S. Affect Detection: An Interdisciplinary Review of Models, Methods, and Their Applications. Affective Computing, IEEE Transactions on 1, 18–37 (2010).

[b5] Petrantonakis P. C. & Hadjileontiadis L. J. Emotion Recognition From EEG Using Higher Order Crossings. Information Technology in Biomedicine, IEEE Transactions on 14, 186–197 (2010). 10.1109/TITB.2009.2034649 19858033

[b6] Ko K.-E., Yang H.-C. & Sim K.-B. Emotion recognition using EEG signals with relative power values and Bayesian network. Int. J. Control Autom. Syst. 7, 865–870 (2009).

[b7] Lisetti C. L. & Nasoz F. Using noninvasive wearable computers to recognize human emotions from physiological signals. Eurasip Journal on Applied Signal Processing 2004, 1672–1687 (2004).

[b8] Picard R. W., Vyzas E. & Healey J. Toward machine emotional intelligence: Analysis of affective physiological state. Ieee Transactions on Pattern Analysis and Machine Intelligence 23, 1175–1191 (2001).

[b9] Collet C., Vernet-Maury E., Delhomme G. & Dittmar A. Autonomic nervous system response patterns specificity to basic emotions. Journal of the Autonomic Nervous System 62, 45–57 (1997). 9021649 10.1016/s0165-1838(96)00108-7

[b10] AlZoubi O., D’Mello S. K. & Calvo R. A. Detecting Naturalistic Expressions of Nonbasic Affect Using Physiological Signals. Affective Computing, IEEE Transactions on 3, 298–310 (2012).

[b11] Valenza G. *et al.* Wearable Monitoring for Mood Recognition in Bipolar Disorder Based on History-Dependent Long-Term Heart Rate Variability Analysis. IEEE Journal of Biomedical and Health Informatics 18, 1625–1635 (2014). 24240031 10.1109/JBHI.2013.2290382

[b12] Camm A. J. *et al.* Heart rate variability - Standards of measurement, physiological interpretation, and clinical use. Circulation 93, 1043–1065 (1996). 8598068

[b13] Chua K. C., Chandran V., Acharya U. R. & Lim C. M. Application of higher order statistics/spectra in biomedical signals-A review. Medical Engineering & Physics 32, 679–689 (2010). 20466580 10.1016/j.medengphy.2010.04.009

[b14] Das M. K. *et al.* Fragmented QRS on a 12-lead ECG: A predictor of mortality and cardiac events in patients with coronary artery disease. Heart Rhythm 4, 1385–1392 (2007). 17954396 10.1016/j.hrthm.2007.06.024

[b15] Nilsson F., Stridh M., Bollmann A. & Sornmo L. Predicting spontaneous termination of atrial fibrillation using the surface ECG. Medical Engineering & Physics 28, 802–808 (2006). 16442328 10.1016/j.medengphy.2005.11.010

[b16] Dilaveris P. E. *et al.* Clinical and electrocardiographic predictors of recurrent atrial fibrillation. PACE-Pacing Clin. Electrophysiol. 23, 352–358 (2000). 10750136 10.1111/j.1540-8159.2000.tb06761.x

[b17] Lin P.-F. *et al.* Correlations between the Signal Complexity of Cerebral and Cardiac Electrical Activity: A Multiscale Entropy Analysis. Plos One 9, 10.1371/journal.pone.0087798 (2014). PMC3912068 24498375

[b18] Rajendra Acharya U. *et al.* Heart rate variability: a review. Med Bio Eng Comput 44, 1031–1051 (2006). 17111118 10.1007/s11517-006-0119-0

[b19] Valenza G. *et al.* Mood states modulate complexity in heartbeat dynamics: A multiscale entropy analysis. Epl. 107, 10.1209/0295-5075/107/18003 (2014).

[b20] Valenza G. *et al.* Revealing Real-Time Emotional Responses: a Personalized Assessment based on Heartbeat Dynamics. Sci. Rep. 4 10.1038/srep04998 (2014). PMC4028901 24845973

[b21] Costa M., Goldberger A. L. & Peng C. K. Multiscale entropy analysis of biological signals. Phys. Rev. E 71, 18 (2005). 10.1103/PhysRevE.71.021906 15783351

[b22] Peng C. K. *et al.* Fractal mechanisms and heart rate dynamics - Long-range correlations and their breakdown with disease. J. Electrocardiol. 28, 59–65 (1995). 8656130 10.1016/s0022-0736(95)80017-4

[b23] Peng C. K., Havlin S., Stanley H. E. & Goldberger A. L. Quantification of scaling exponents and crossover phenomena in nonstationary heartbeat time-series. Chaos 5, 82–87 (1995). 11538314 10.1063/1.166141

[b24] Robinson P. A. *et al.* Prediction of electroencephalographic spectra from neurophysiology. Phys. Rev. E 63, 18 (2001). 10.1103/PhysRevE.63.021903 11308514

[b25] Cantero J. L., Atienza M., Gomez C. M. & Salas R. M. Spectral structure and brain mapping of human alpha activities in different arousal states. Neuropsychobiology 39, 110–116 (1999). 10072668 10.1159/000026569

[b26] Rodriguez E. *et al.* Perception’s shadow: long-distance synchronization of human brain activity. Nature 397, 430–433 (1999). 9989408 10.1038/17120

[b27] Bertrand O. & Tallon-Baudry C. Oscillatory gamma activity in humans: a possible role for object representation. Int. J. Psychophysiol. 38, 211–223 (2000). 11102663 10.1016/s0167-8760(00)00166-5

[b28] Laufs H. *et al.* EEG-correlated fMRI of human alpha activity. Neuroimage 19, 1463–1476 (2003). 12948703 10.1016/s1053-8119(03)00286-6

[b29] Thayer J. F. & Lane R. D. Claude Bernard and the heart–brain connection: Further elaboration of a model of neurovisceral integration. Neuroscience & Biobehavioral Reviews 33, 81–88 (2009). 18771686 10.1016/j.neubiorev.2008.08.004

[b30] Quintana D. S. *et al.* Heart rate variability is associated with emotion recognition: Direct evidence for a relationship between the autonomic nervous system and social cognition. Int. J. Psychophysiol. 86, 168–172 (2012). 22940643 10.1016/j.ijpsycho.2012.08.012

[b31] Tang S.-C. *et al.* Complexity of heart rate variability predicts outcome in intensive care unit admitted patients with acute stroke. Journal of Neurology Neurosurgery and Psychiatry 86, 95–100 (2015). 10.1136/jnnp-2014-308389 25053768

[b32] Ho Y.-L., Lin C., Lin Y.-H. & Lo M.-T. The Prognostic Value of Non-Linear Analysis of Heart Rate Variability in Patients with Congestive Heart Failure-A Pilot Study of Multiscale Entropy. Plos One 6, 10.1371/journal.pone.0018699 (2011). PMC3076441 21533258

[b33] Basar E. & Guntekin B. A breakthrough in neuroscience needs a “Nebulous Cartesian System” Oscillations, quantum dynamics and chaos in the brain and vegetative system. Int. J. Psychophysiol. 64, 108–122 (2007). 17049654 10.1016/j.ijpsycho.2006.07.012

[b34] Palva S. & Palva J. M. New vistas for alpha-frequency band oscillations. Trends in Neurosciences 30, 150–158 (2007). 17307258 10.1016/j.tins.2007.02.001

[b35] Kober H. *et al.* Functional grouping and cortical-subcortical interactions in emotion: A meta-analysis of neuroimaging studies. Neuroimage 42, 998–1031 (2008). 18579414 10.1016/j.neuroimage.2008.03.059 PMC2752702

[b36] Lim Y.-H. *et al.* Effect of diurnal temperature range on cardiovascular markers in the elderly in Seoul, Korea. International Journal of Biometeorology 57, 597–603 (2013). 22956153 10.1007/s00484-012-0587-x

[b37] Costa M. *et al.* Noise and poise: Enhancement of postural complexity in the elderly with a stochastic-resonance-based therapy. Europhysics letters 77, 68008 (2007). 17710211 10.1209/0295-5075/77/68008 PMC1949396

[b38] Uusberg A., Uibo H., Kreegipuu K. & Allik J. EEG alpha and cortical inhibition in affective attention. Int. J. Psychophysiol. 89, 26–36 (2013). 23643563 10.1016/j.ijpsycho.2013.04.020

[b39] Richman J. S. & Moorman J. R. Physiological time-series analysis using approximate entropy and sample entropy. American journal of physiology 278, H2039–H2049 (2000). 10843903 10.1152/ajpheart.2000.278.6.H2039

[b40] Huikuri H. V. & Mäkikallio T. H. Heart rate variability in ischemic heart disease. Autonomic Neuroscience 90, 95–101 (2001). 11485298 10.1016/S1566-0702(01)00273-9

[b41] Bigan C. & Woolfson M. S. Time-frequency analysis of short segments of biomedical data. Science, Measurement and Technology, IEE Proceedings 147, 368–373 (2000).

[b42] Durka P. J. *et al.* On the statistical significance of event-related EEG desynchronization and synchronization in the time-frequency plane. Biomedical Engineering, IEEE Transactions on 51, 1167–1175 (2004). 10.1109/TBME.2004.827341 15248533

[b43] Gloor P. WORK OF BERGER, H. Electroencephalography and Clinical Neurophysiology 27, 649 (1969). 4187257 10.1016/0013-4694(69)91207-3

[b44] Werts C. E. & Linn R. L. A general linear model for studying growth. Psychological Bulletin 73, 17–22 (1970).

[b45] Hastie T. J. & Tibshirani R. J. Generalized Additive Models. Ch. 6, 136–174 (Taylor & Francis, 1990).

