# Supplementary material for: Complexity of cardiac signals for predicting changes in alpha-waves after stress in patients undergoing cardiac catheterization
Source: Sci Rep. 2015 Aug 19;5:13315. doi: 10.1038/srep13315 (PMC4541158; doi:10.1038/srep13315)
Supplement: Supplementary Information [file srep13315-s1.pdf]

Complexity of cardiac signals for predicting changes in alpha-waves after stress in patients undergoing cardiac catheterization

Supplementary Information

Hung-Chih Chiu[1,]*, Yen-Hung Lin[2,]*, Men-Tzung Lo[3,4], Sung-Chun Tang[6], Tzung-Dau Wang[2], Hung-Chun Lu[2], Yi-Lwun Ho[2], Hsi-Pin Ma[1] & Chung-Kang Peng[4,5]

[1]Department of Electrical Engineering, National Tsing Hua University, Hsinchu, Taiwan.

[2]Department of Internal Medicine, National Taiwan University Hospital and National Taiwan University College of Medicine, Taipei, Taiwan.

[3]Institute of Translational and Interdisciplinary Medicine and Department of Biomedical Sciences and Engineering, National Central University, Chungli, Taiwan

[4]Research Center for Adaptive Data Analysis, National Central University, Taoyuan, Taiwan.

[5]Division of Interdisciplinary Medicine and Biotechnology, Beth Israel Deaconess Medical Center/Harvard Medical School, Boston, Massachusetts, USA.

[6]Department of Neurology, National Taiwan University Hospital and National Taiwan University College of Medicine, Taipei, Taiwan.

Supplementary Table S1 | Classification of patients on the basis of the pretest variables and the difference between pre- and post-test variables.

ECG	Linear Variable	Pre-Test	meanNN, Log-meanNN,<br>sdNN, Log-sdNN,<br>pNN20, Log-pNN20,<br>pNN50, Log-pNN50,<br>rMMSD, Log-rMMSD,<br>LF, Log-LF, HF, Log-HF<br>LF/HF, Log-LF/HF
		Difference	meanNN, Log-meanNN,<br>sdNN, Log-sdNN,<br>pNN20, Log-pNN20,<br>pNN50, Log-pNN50,<br>rMMSD, Log-rMMSD,<br>LF, Log-LF, HF, Log-HF<br>LF/HF, Log-LF/HF
	Non-linear Variable	Pre-Test	Slope1-5, Slope6-20,<br>Area1-5, Log_Area1-5,<br>Area6-20, Log_Area6-20<br>$\propto_1$, Log_ $\propto_1$, $\propto_2$, Log_ $\propto_2$
		Difference	Slope1-5, Slope6-20,<br>Area1-5, Log_Area1-5,<br>Area6-20, Log_Area6-20<br>$\propto_1$, Log-$\propto_1$, $\propto_2$, Log- $\propto_2$
EEG	Linear Variable	Pre-Test	Alpha activity
		Difference	Alpha activity

Clinical Data	Age, Gender, BMI, MBP(Pre-Test), MBP(Difference), Treatment, Smoking, Diabetes Mellitus, Hyperlipidemia, Hypertension, EGFR
Neurotransmitter	Serotonin, Orphanin_FQ, Dopamine

Supplementary Table S2 | Classification of patients according to Spearman's rank correlation.

ECG	Linear Variable	Pre-Test	meanNN,<br>sdNN, Log-sdNN,<br>pNN20, Log-pNN20,<br>LF/HF, Log-LF/HF
		Difference	meanNN,<br>pNN20, Log_pNN20,<br>rMMSD,<br>LF/HF, Log_LF/HF
	Non-linear Variable	Pre-Test	Slope1-5,<br>Area1-5, Log-Area1-5,<br>$\propto_1$, $\propto_2$
		Difference	Slope1-5,<br>Area1-5, Log-Area1-5,<br>$\propto_1$, $\propto_2$
EEG	Linear Variable	Pre-Test	Alpha activity
		Difference	Alpha activity
Clinical Data	Age, Gender, BMI, MBP(Pre-Test), MBP(Difference), Treatment, Smoking, Diabetes Mellitus, Hyperlipidemia, Hypertension, estimated glomerular filtration rate		
Neurotransmitter			Serotonin, Orphanin_FQ, Dopamine

Supplementary Table S3 | Classification of methods by using GAMs.

ECG	Linear Variable	Pre-Test	$3.816 \leq Log\_sdNN \leq 5.115$,<br>$0.017 \leq pNN50 \leq 0.176$,<br>$56.598 \leq rMSSD \leq 194.443$,<br>$3.81 \leq Log\_rMSSD \leq 5.348$,<br>$1071.873 \leq HF \leq 13485.712$,<br>$1.229 \leq LF/HF \leq 1.98$,
		Difference	$Log\_sdNN \leq -0.415$,<br>$-0.164 \leq pNN20 \leq 0.007$,<br>$-0.142 \leq pNN50 \leq 0.007$,<br>$-105.641 \leq rMSSD \leq 4.677$,<br>$LF \leq 156.425$ and $LF \geq 13413.356$,<br>$HF \leq 376.933$ and $HF \geq 25327.388$
	Non-linear Variable	Pre-Test	$0.01 \leq Slope6-20$,<br>$Log\_Area1-5 \leq 1.007$,<br>$2.183 \leq Log\_Area6-20 \leq 2.895$,<br>$0.425 \leq \propto_1 \leq 0.756$,<br>$\propto_2 \leq 0.737$ and $\propto_2 \geq 0.876$
		Difference	$Area1-5 \leq -1.247$ and $Area1-5 \geq 0.809$,<br>$Log\_Area6-20 \leq -0.042$ and $Log\_Area6-20 \geq 0.5$

Supplementary Table S4 | Experimental results of the HRV analysis before *treatment, obtained using S*pearman's rank correlation.

	sdNN	Log-sdNN	pNN50	Log-pNN50	rMMSD	Log-rMMSD	LF	Log-LF	LF/HF	Log-LF/HF
**pNN50**	0.9157	0.9157								
**Log- pNN50**	0.9157	0.9157								
**rMMSD**	0.9129	0.9129	0.8638	0.8638						
**Log-rMMSD**	0.9129	0.9129	0.8638	0.8638						
**LF**	0.8364	0.8364			0.845	0.845				
**Log-LF**	0.8364	0.8364			0.845	0.845				
**HF**	0.8151	0.8151			0.9092	0.9092	0.9487	0.9487		
**Log-HF**	0.8151	0.8151			0.9092	0.9092	0.9487	0.9487		
$\propto_1$									0.8331	0.8331
**Log--**$\propto_1$									0.8331	0.8331

Supplementary Table S5 | Experimental results regarding the difference between the pre- and posttest variables obtained using Spearman's rank correlation

	sdNN	Log-sdNN	LF	Log-LF	LF/HF
**rMMSD**	0.8979	0.8564			
**Log- rMMSD**	0.8320	0.8760			
**HF**			0.9486		
**Log-HF**				0.9405	
**Log-LF/HF**					0.9782

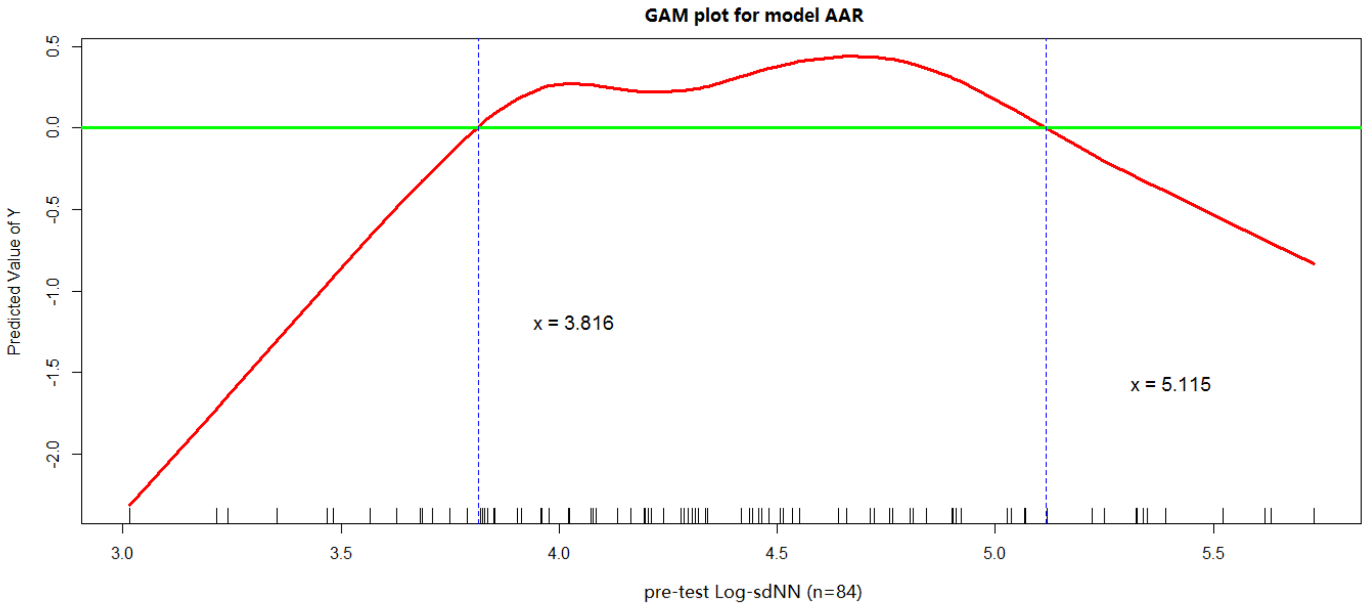

Supplementary Figure S1: GAM plot for modeling the relationship between the change in the alpha wave and pretest log-sdNN. The vertical axis represents the difference in alpha activity. The horizontal axis represents the pretest log-sdNN and number of patients. GAM techniques can be used to show that

$$\text{pre-test Log-sdNN} = \begin{cases} 1, & 3.816 \leq \text{Log-sdNN} \leq 5.115 \\ 0, & \text{others} \end{cases}$$

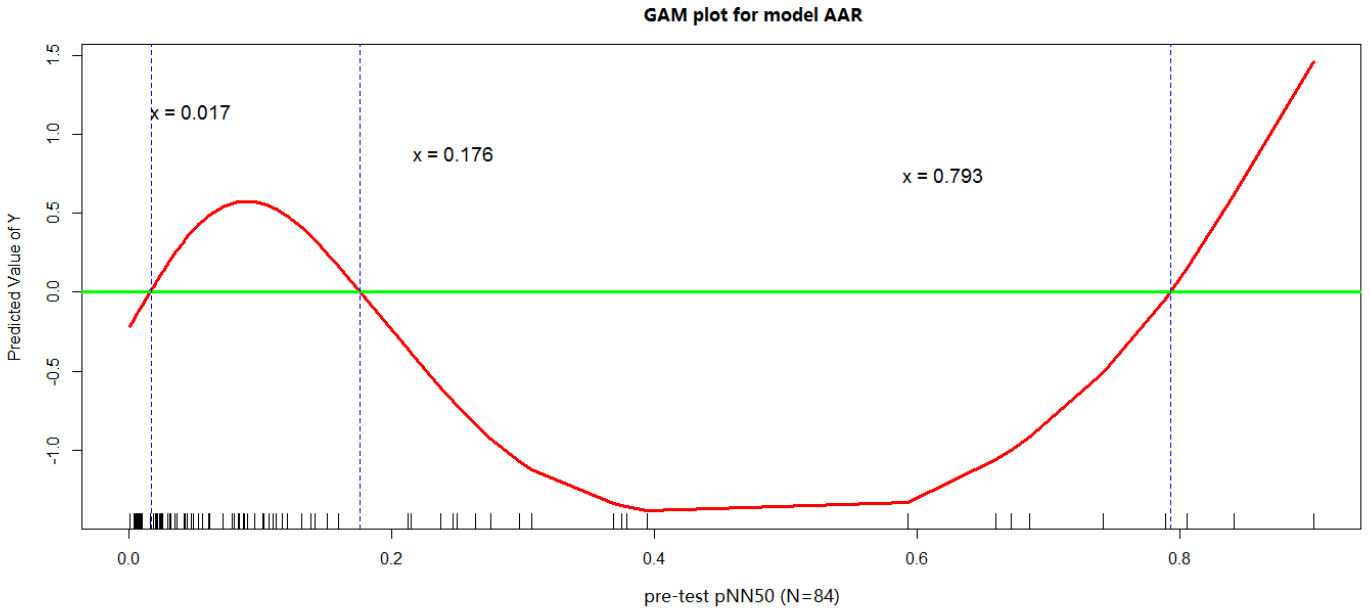

Supplementary Figure S2: GAM plot for modeling the relationship between the change in the alpha wave and pretest pNN50. The vertical axis represents the difference in alpha activity. The horizontal axis represents the pretest pNN50 and number of patients. GAM techniques can be used to show that

$$\text{pre-test pNN50} = \begin{cases} 1, & 0.017 \leq \text{pNN50} \leq 0.176 \\ 0, & \text{others} \end{cases}$$

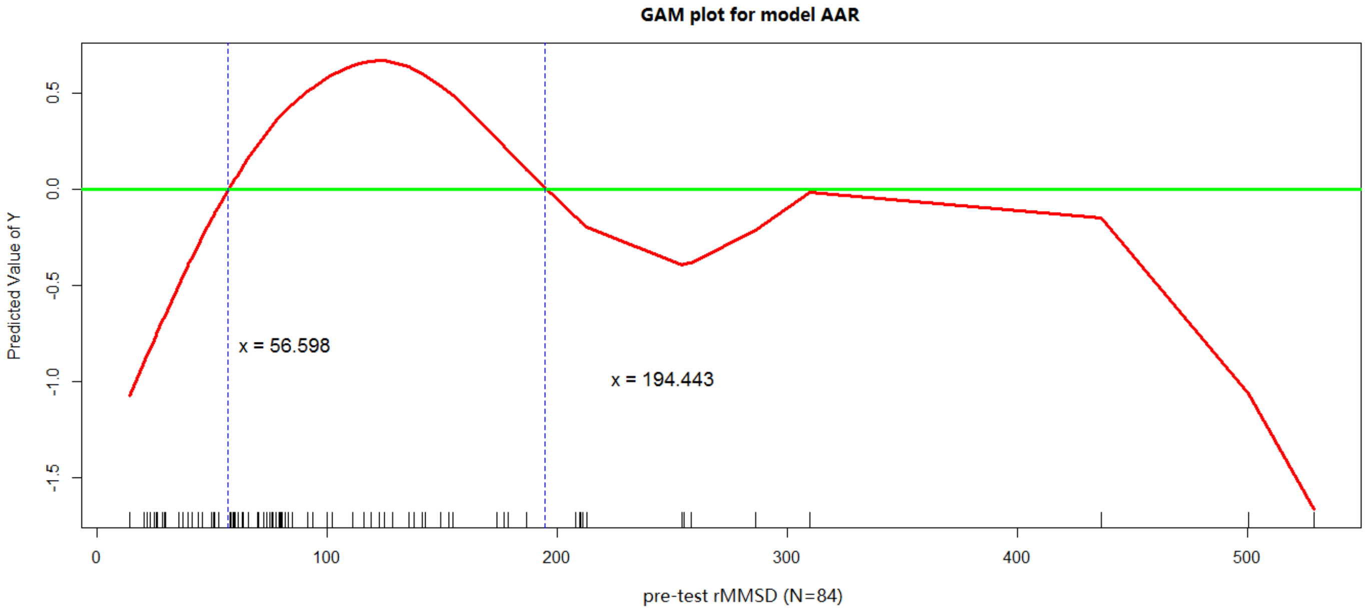

Supplementary Figure S3: GAM plot for modeling the relationship between the change in the alpha wave and pretest rMMSD. The vertical axis represents the difference in alpha activity. The horizontal axis represents the pretest rMMSD and number of patients. GAM techniques can be used to show that

$$\text{pre-test rMMSD} = \begin{cases} 1, & 56.598 \leq \text{rMMSD} \leq 194.443 \\ 0, & \text{others} \end{cases}$$

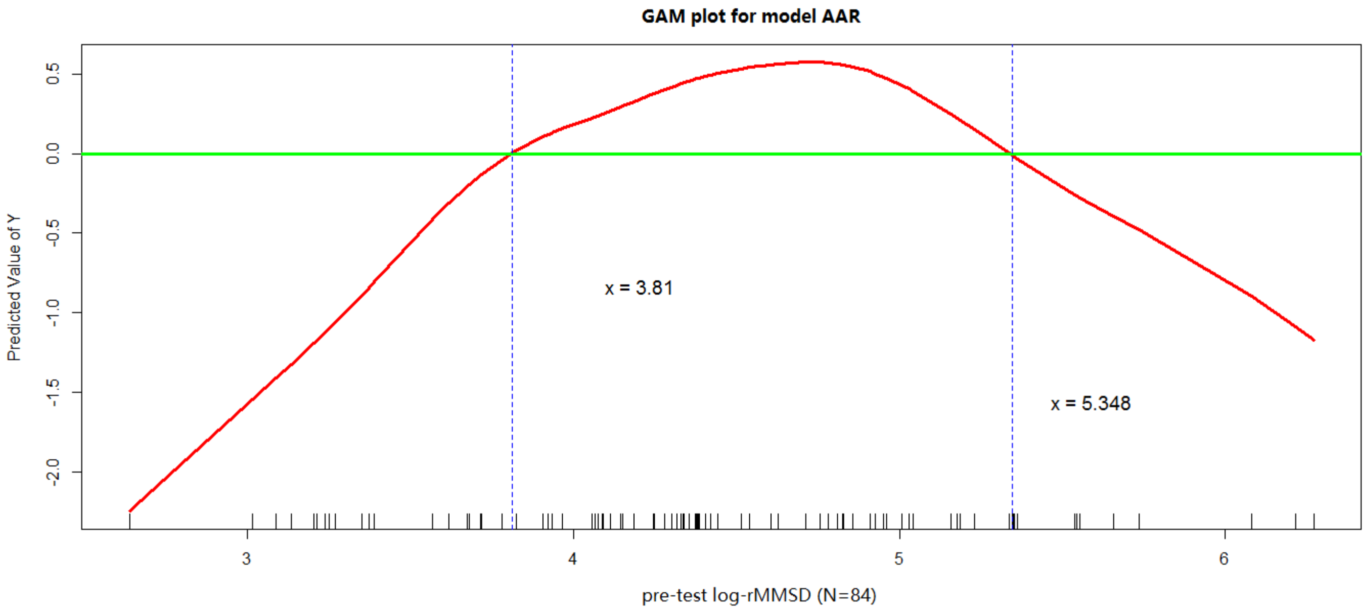

Supplementary Figure S4: GAM plot for modeling the relationship between the change in the alpha wave and pretest log-rMMSD. The vertical axis represents the difference in alpha activity. The horizontal axis represents the pretest log-rMMSD and number of patients. GAM techniques can be used to show that

$$\text{pre-test log-rMMSD} = \begin{cases} 1, & 3.81 \leq \text{log-rMMSD} \leq 5.348 \\ 0, & \text{others} \end{cases}$$

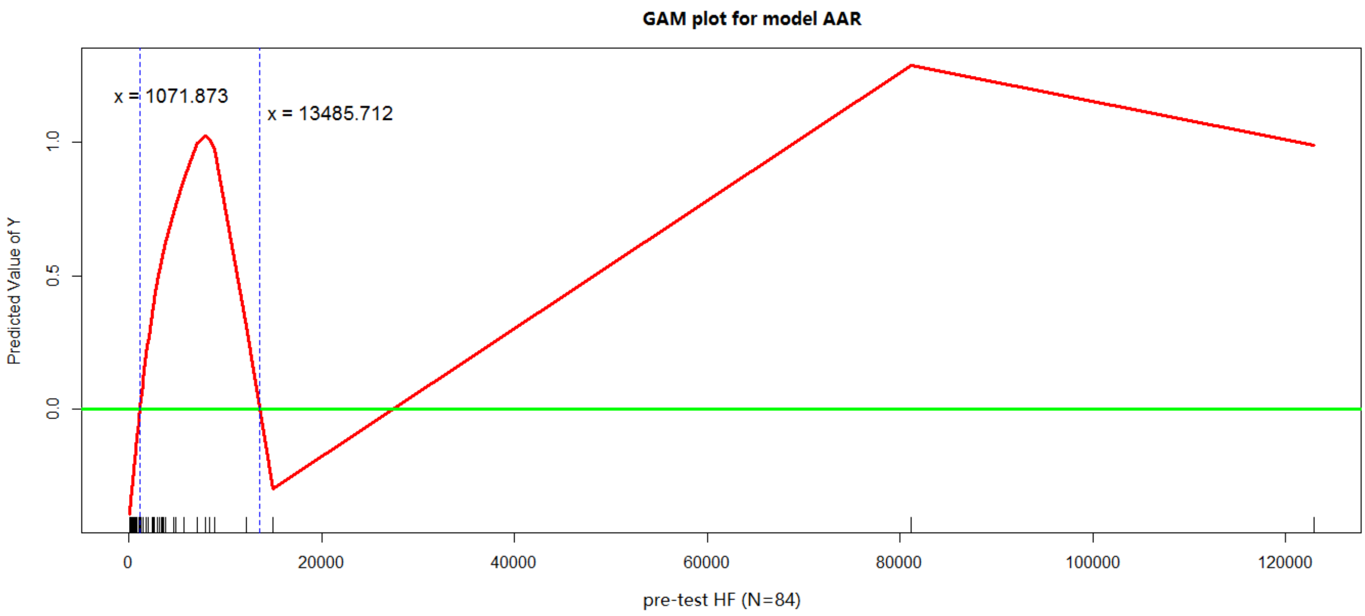

Supplementary Figure S5: GAM plot for modeling the relationship between the change in the alpha wave and pretest HF. The vertical axis represents the difference in alpha activity. The horizontal axis represents the pretest HF and number of patients. GAM techniques can be used to show that

$$\text{pre-test HF} = \begin{cases} 1, & 1071.873 \leq \text{HF} \leq 13485.712 \\ 0, & \text{others} \end{cases}$$

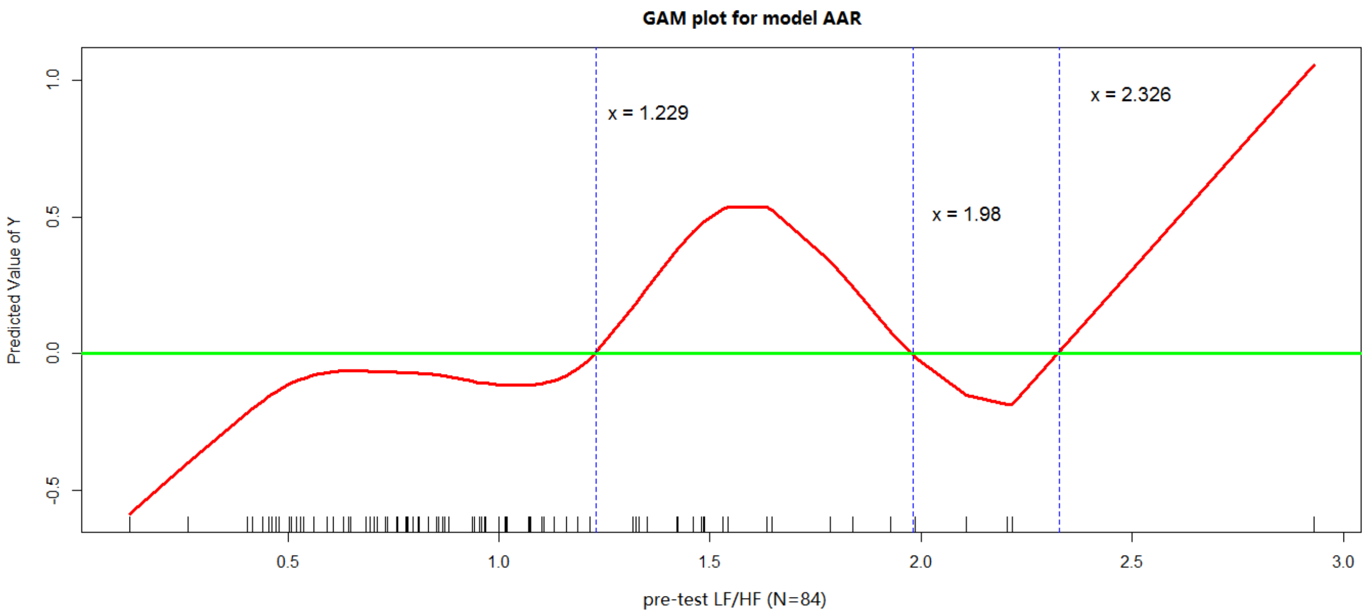

Supplementary Figure S6: GAM plot for modeling the relationship between the change in the alpha wave and pretest LF/HF. The vertical axis represents the difference in alpha activity. The horizontal axis represents the pretest LF/HF and number of patients. GAM techniques can be used to show that

$$\text{pre-test LF/HF} = \begin{cases} 1, & 1.229 \leq \text{LF/HF} \leq 1.98 \\ 0, & \text{others} \end{cases}$$

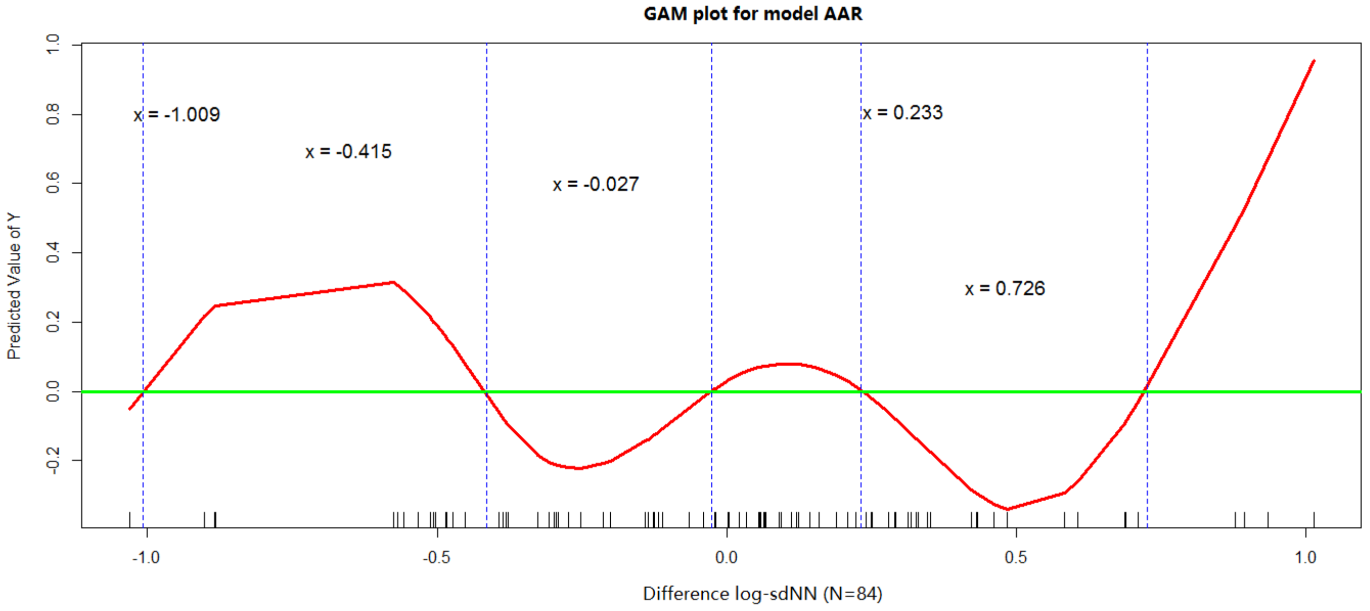

Supplementary Figure S7: GAM plot for modeling the relationship between the change in the alpha wave and Difference log-sdNN. The vertical axis represents the difference in alpha activity. The horizontal axis represents the Difference log-sdNN and number of patients. GAM techniques can be used to show that

$$\text{Difference log-sdNN} = \begin{cases} 1, & \text{log-sdNN} \leq -0.415 \\ 0, & \text{others} \end{cases}$$

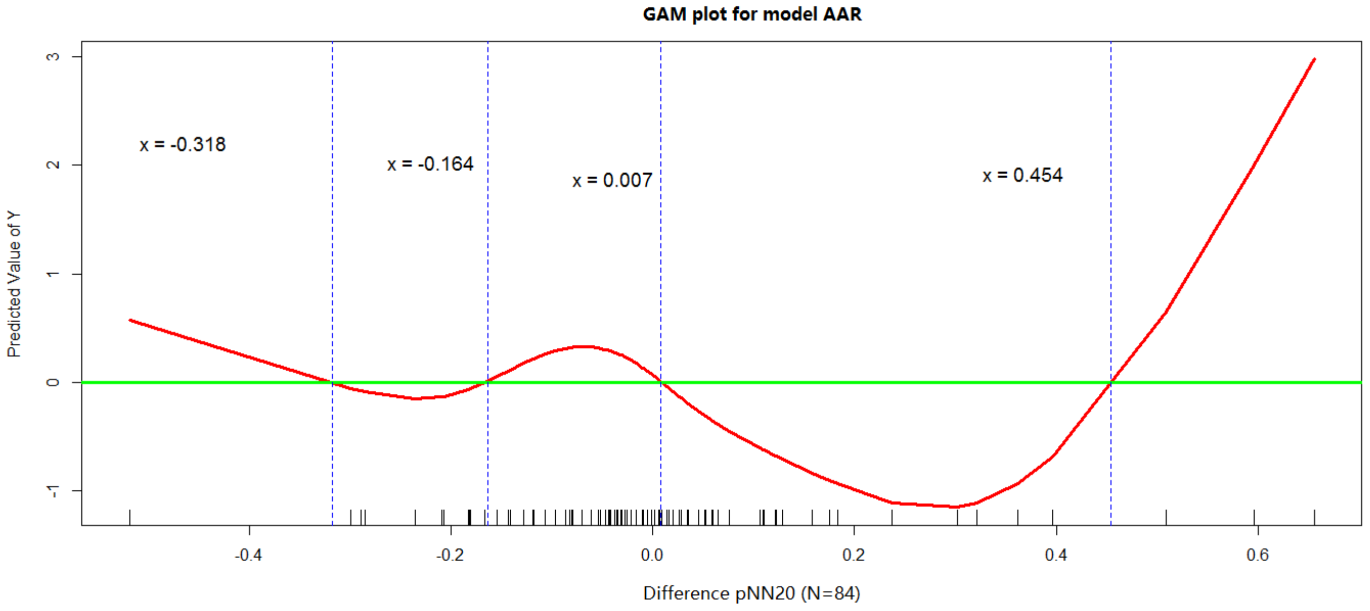

Supplementary Figure S8: GAM plot for modeling the relationship between the change in the alpha wave and Difference pNN20. The vertical axis represents the difference in alpha activity. The horizontal axis represents the Difference pNN20 and number of patients. GAM techniques can be used to show that

$$\text{Difference pNN20} = \begin{cases} 1, & -0.164 \leq \text{pNN20} \leq 0.007 \\ 0, & \text{others} \end{cases}$$

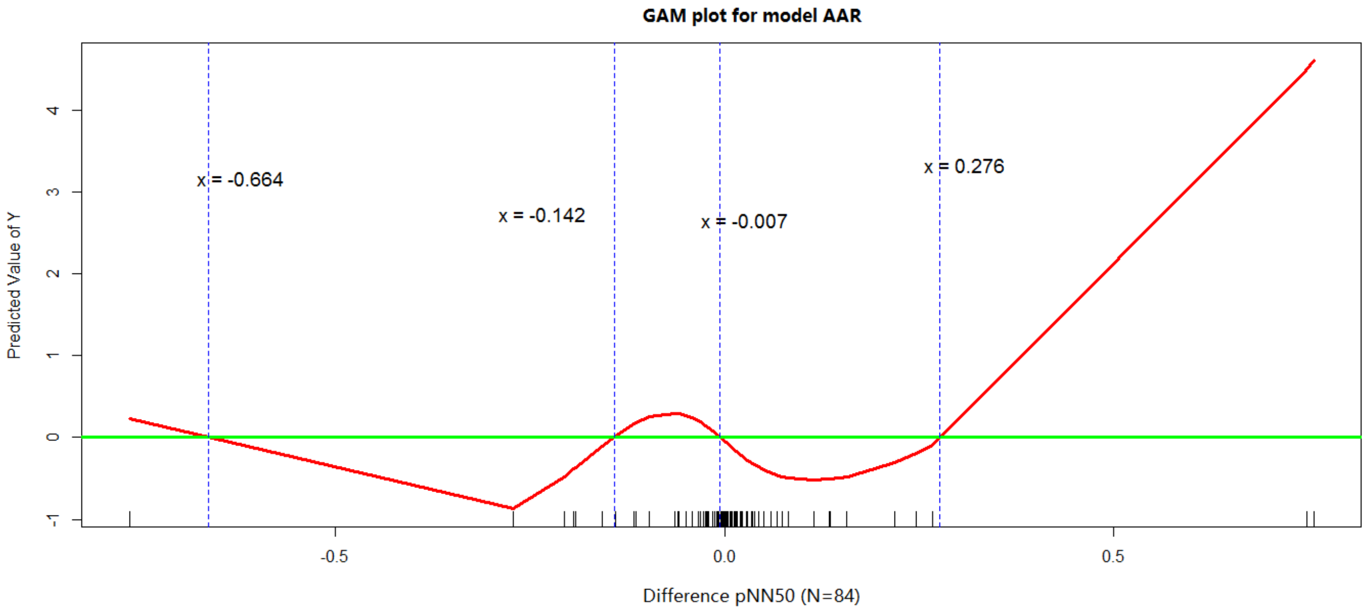

Supplementary Figure S9: GAM plot for modeling the relationship between the change in the alpha wave and Difference pNN50. The vertical axis represents the difference in alpha activity. The horizontal axis represents the Difference pNN50 and number of patients. GAM techniques can be used to show that

$$\text{Difference pNN50} = \begin{cases} 1, & -0.142 \leq \text{pNN50} \leq 0.007 \\ 0, & \text{others} \end{cases}$$

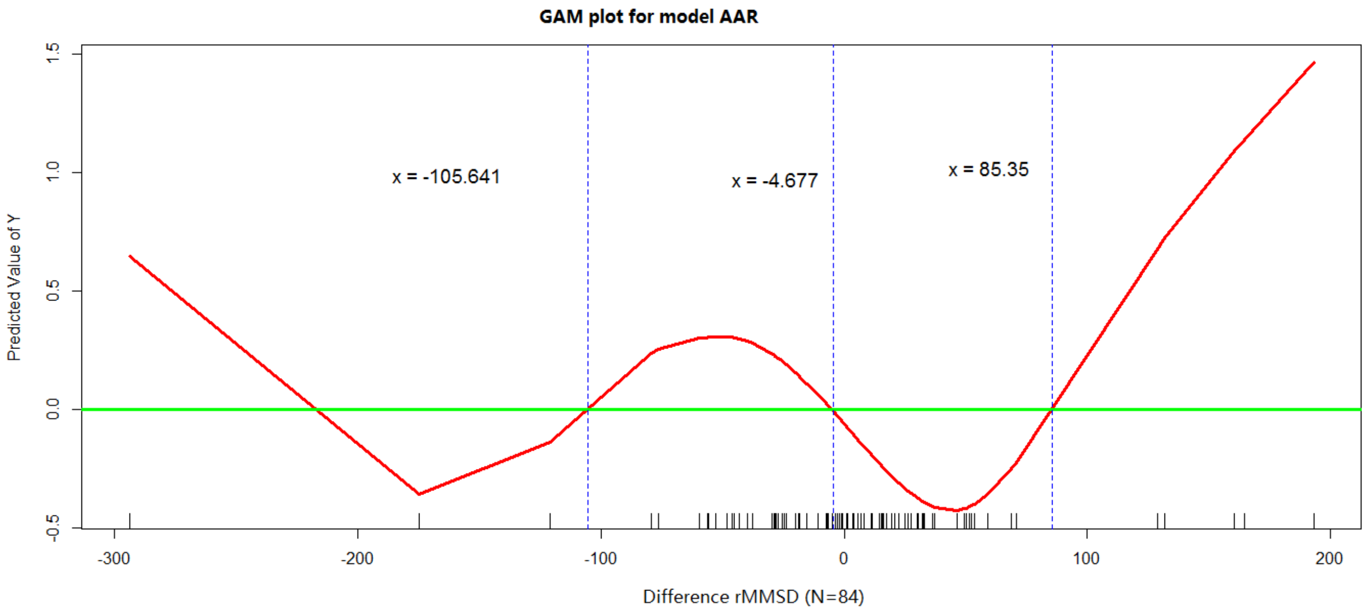

Supplementary Figure S10: GAM plot for modeling the relationship between the change in the alpha wave and Difference rMMSD. The vertical axis represents the difference in alpha activity. The horizontal axis represents the Difference rMMSD and number of patients. GAM techniques can be used to show that

$$\text{Difference rMMSD} = \begin{cases} 1, & -105.641 \leq \text{rMMSD} \leq 4.677 \\ 0, & \text{others} \end{cases}$$

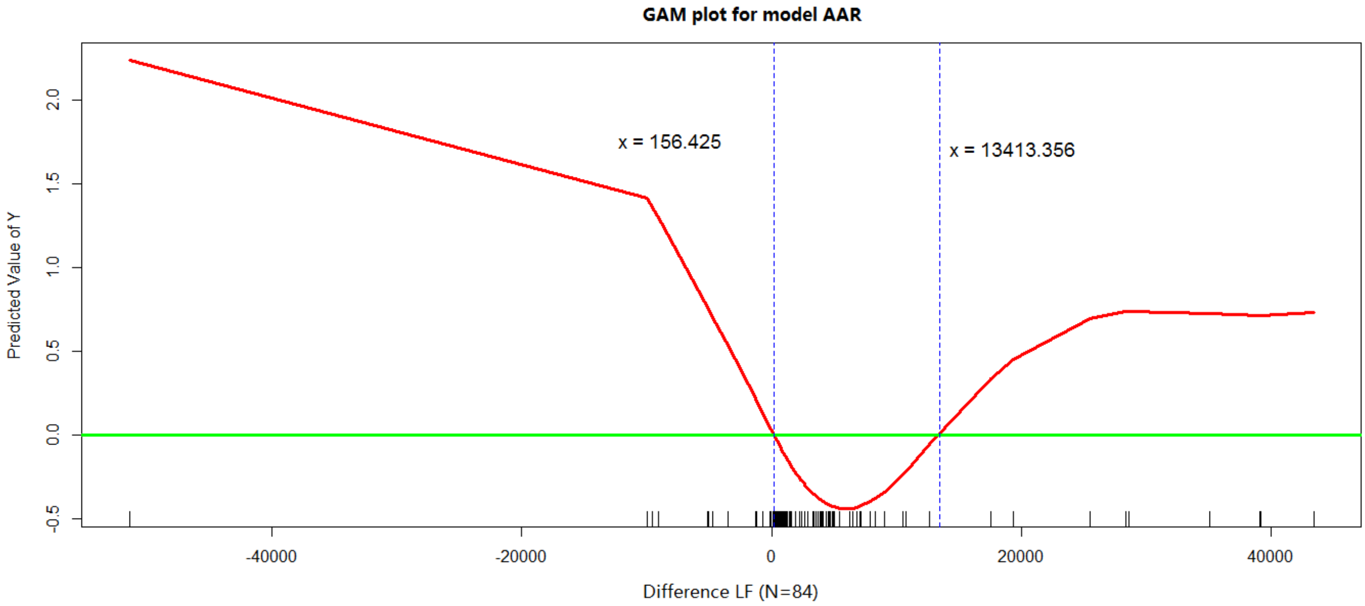

Supplementary Figure S11: GAM plot for modeling the relationship between the change in the alpha wave and Difference LF. The vertical axis represents the difference in alpha activity. The horizontal axis represents the Difference LF and number of patients. GAM techniques can be used to show that

$$\text{Difference LF} = \begin{cases} 1, & 156.425 \leq \text{LF} \leq 13413.356 \\ 0, & \text{others} \end{cases}$$

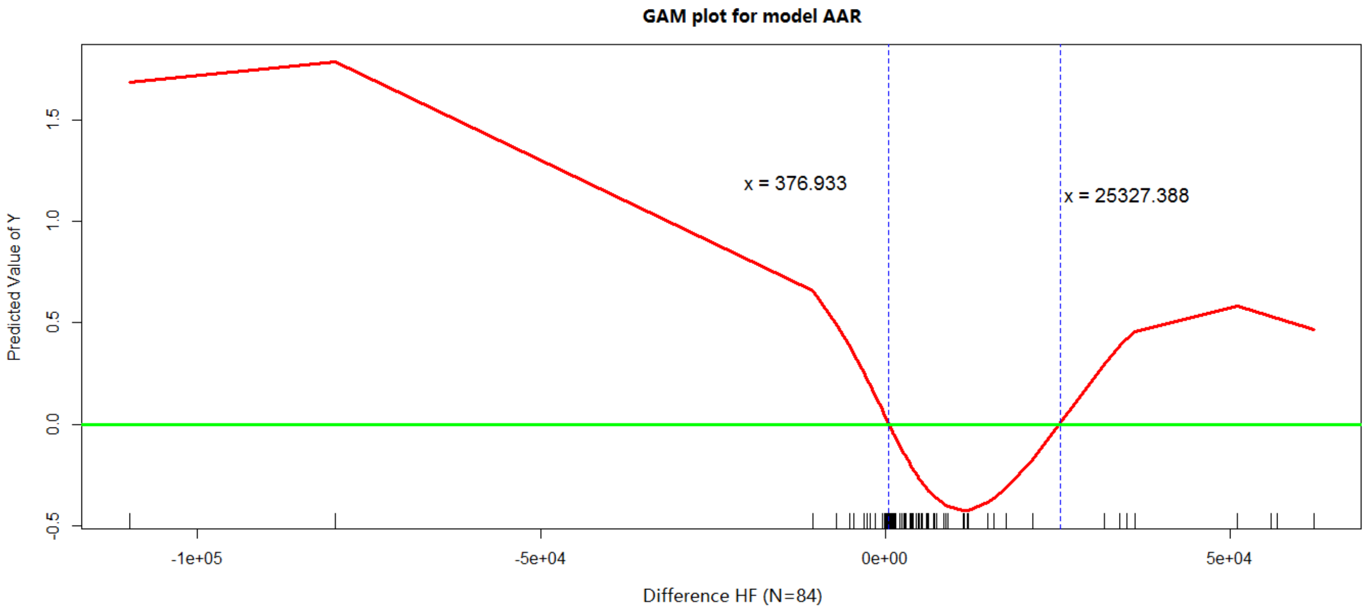

Supplementary Figure S12: GAM plot for modeling the relationship between the change in the alpha wave and Difference HF. The vertical axis represents the difference in alpha activity. The horizontal axis represents the Difference HF and number of patients. GAM techniques can be used to show that

$$\text{Difference HF} = \begin{cases} 1, & 376.933 \leq \text{HF} \leq 25327.388 \\ 0, & \text{others} \end{cases}$$

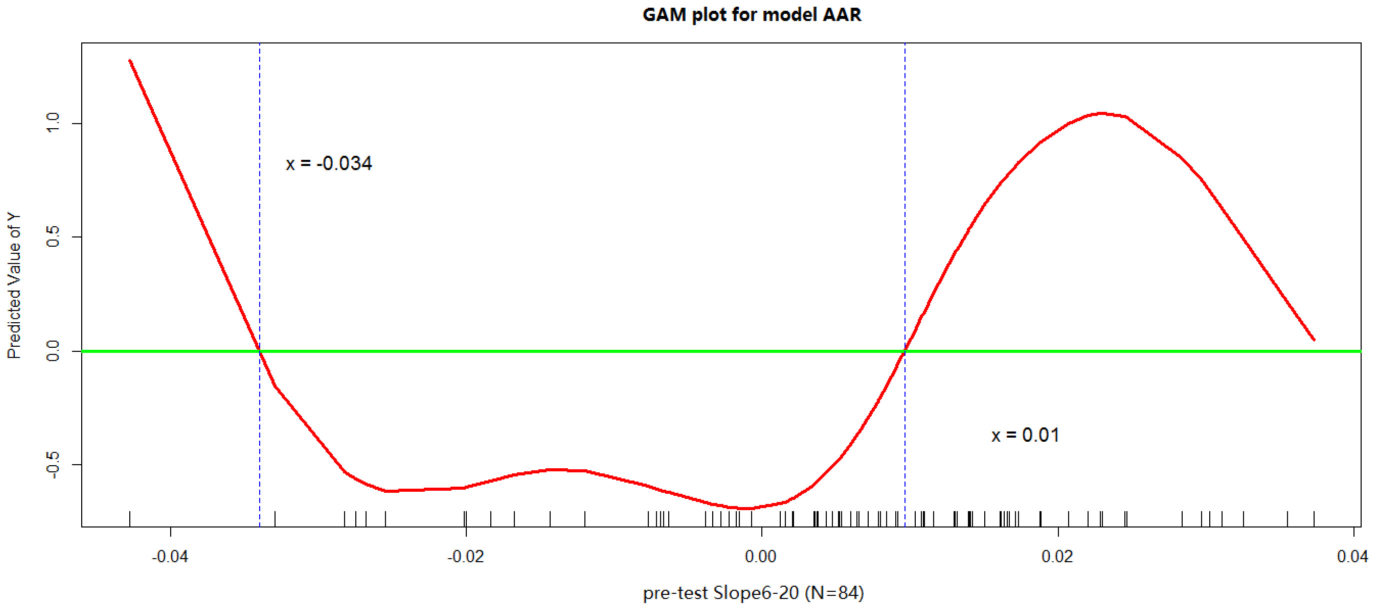

Supplementary Figure S13: GAM plot for modeling the relationship between the change in the alpha wave and pre-test Slope6-20. The vertical axis represents the difference in alpha activity. The horizontal axis represents the pretest Slope6-20and number of patients. GAM techniques can be used to show that

$$\text{pre-test Slope6-20} = \begin{cases} 1, & 0.01 \leq \text{Slope6-20} \\ 0, & \text{others} \end{cases}$$

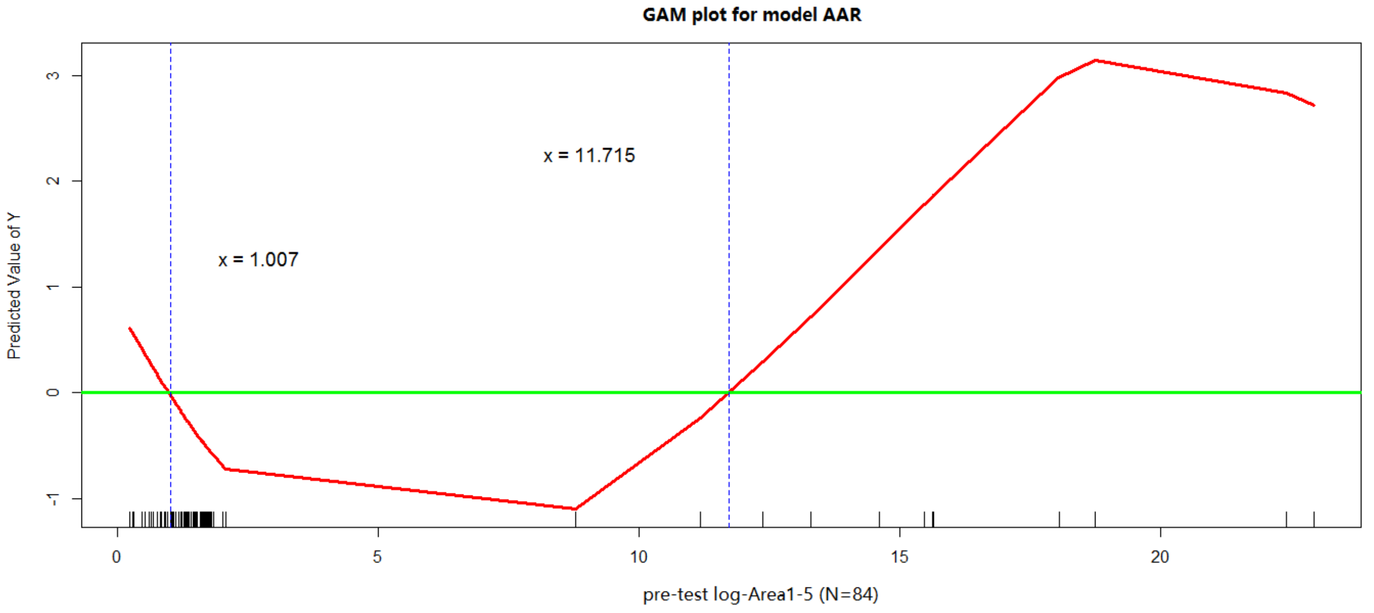

Supplementary Figure S14: GAM plot for modeling the relationship between the change in the alpha wave and pre-test log-Area1-5. The vertical axis represents the difference in alpha activity. The horizontal axis represents the pretest log-Area1-5and number of patients. GAM techniques can be used to show that

$$\text{pre-test log-Area1-5} = \begin{cases} 1, & \text{log-Area1-5} \leq 1.007 \\ 0, & \text{others} \end{cases}$$

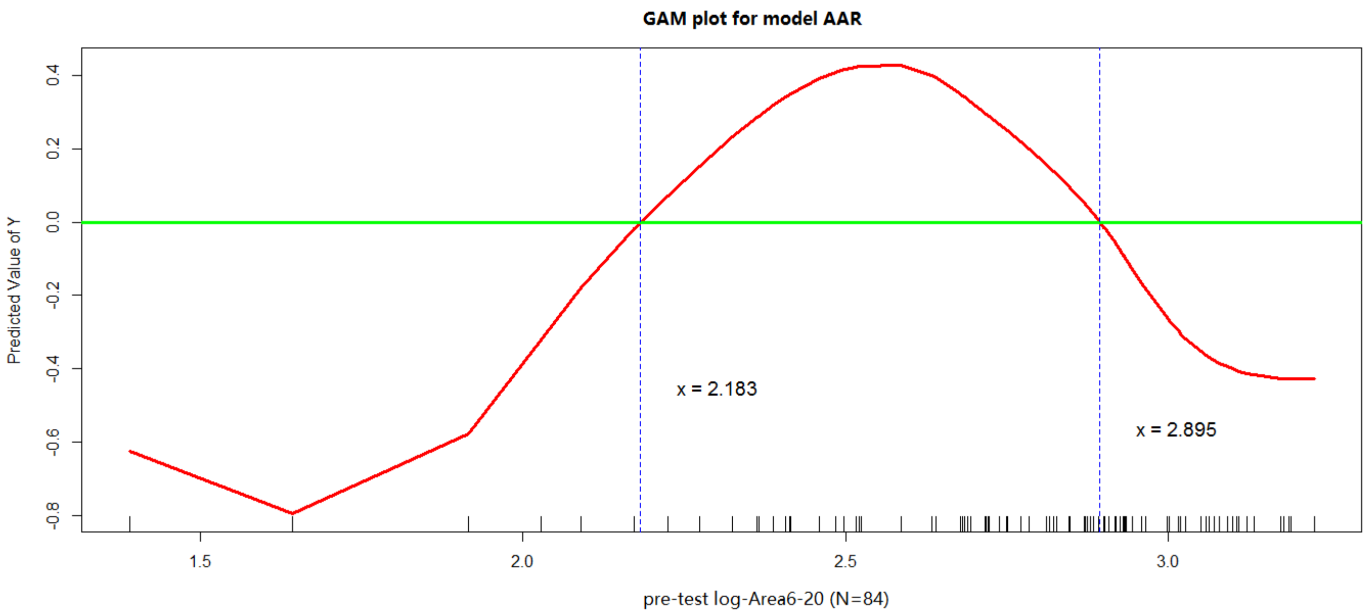

Supplementary Figure S15: GAM plot for modeling the relationship between the change in the alpha wave and pre-test log-Area6-20. The vertical axis represents the difference in alpha activity. The horizontal axis represents the pretest log-Area6-20 and number of patients. GAM techniques can be used to show that

$$\text{pre-test log-Area6-20} = \begin{cases} 1, & 2.183 \leq \text{log-Area6-20} \leq 2.895 \\ 0, & \text{others} \end{cases}$$

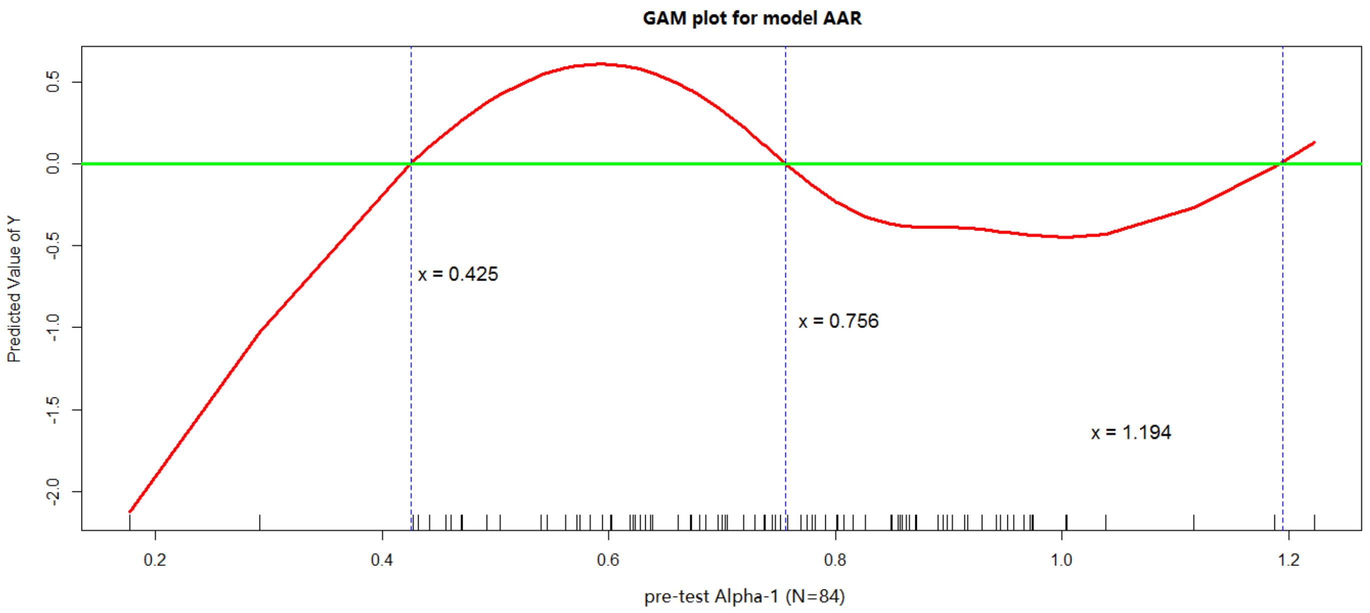

Supplementary Figure S16: GAM plot for modeling the relationship between the change in the alpha wave and pre-test Alpha-1. The vertical axis represents the difference in alpha activity. The horizontal axis represents the pretest Alpha-1 and number of patients. GAM techniques can be used to show that

$$\text{pre-test Alpha-1} = \begin{cases} 1, & 0.425 \leq \text{Alpha-1} \leq 0.756 \\ 0, & \text{others} \end{cases}$$

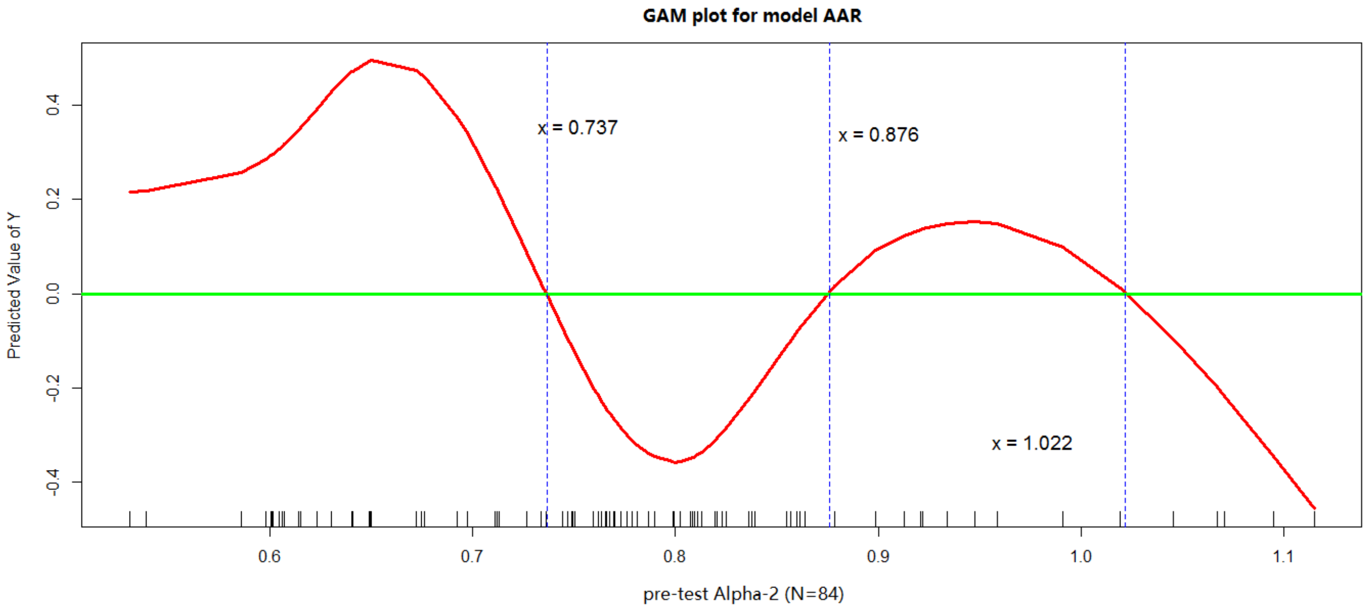

Supplementary Figure S17: GAM plot for modeling the relationship between the change in the alpha wave and pre-test Alpha-2. The vertical axis represents the difference in alpha activity. The horizontal axis represents the pretest Alpha-2 and number of patients. GAM techniques can be used to show that

$$\text{pre-test Alpha-2} = \begin{cases} 1, & \text{Alpha-2} \leq 0.737 \text{ and Alpha-2} \geq 0.876 \\ 0, & \text{others} \end{cases}$$

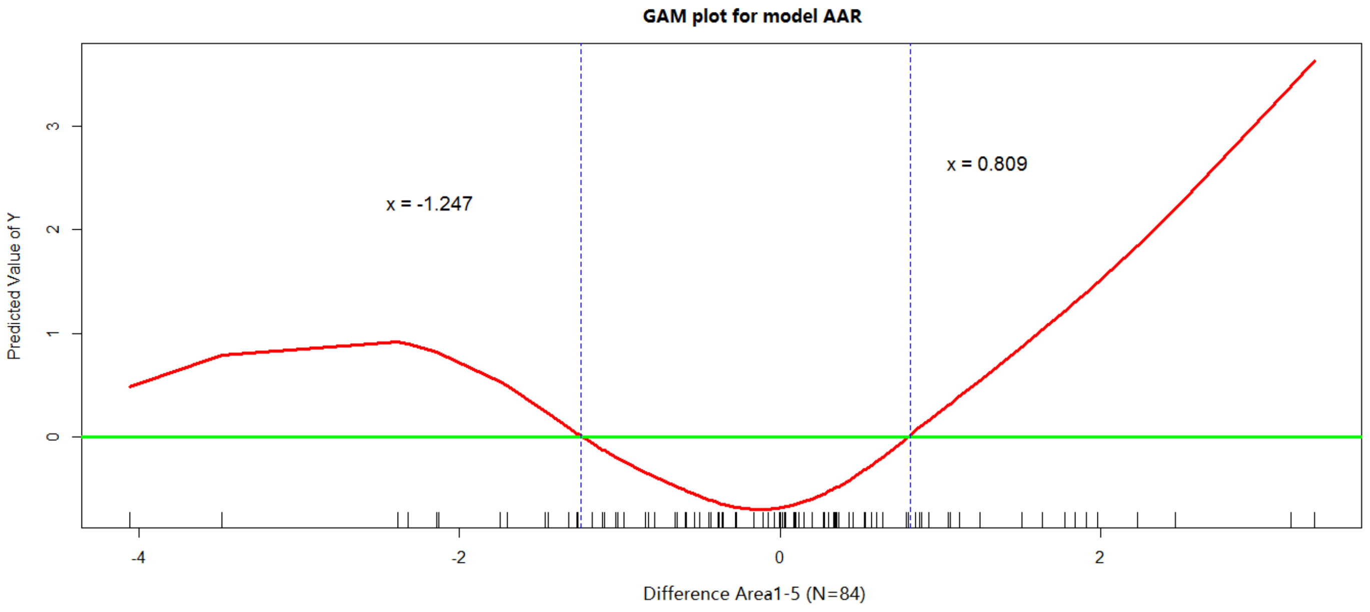

Supplementary Figure S18: GAM plot for modeling the relationship between the change in the alpha wave and Difference Area1-5. The vertical axis represents the difference in alpha activity. The horizontal axis represents the Difference Area1-5 and number of patients. GAM techniques can be used to show that

$$\text{Difference Area1-5} = \begin{cases} 1, & \text{Area1-5} \leq -1.247 \text{ and Area1-5} \geq 0.809 \\ 0, & \text{others} \end{cases}$$

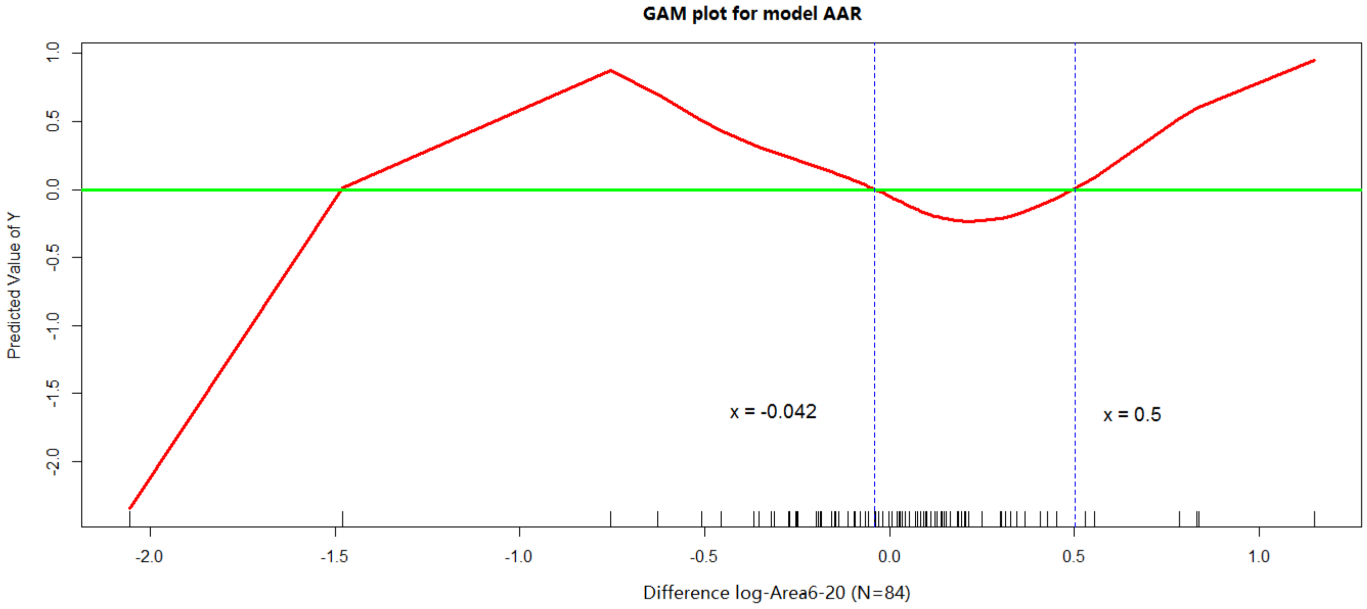

Supplementary Figure S19: GAM plot for modeling the relationship between the change in the alpha wave and Difference log-Area6-20. The vertical axis represents the difference in alpha activity. The horizontal axis represents the Difference log-Area6-20 and number of patients. GAM techniques can be used to show that

$$\text{Difference log-Area6-20} = \begin{cases} 1, & \text{Area6-20} \leq -0.042 \text{ and Area6-20} \geq 0.5 \\ 0, & \text{others} \end{cases}$$
